# Interferon gamma mediates the reduction of adipose tissue regulatory T cells in human obesity

**DOI:** 10.1038/s41467-022-33067-5

**Published:** 2022-09-24

**Authors:** David Bradley, Alan J. Smith, Alecia Blaszczak, Dharti Shantaram, Stephen M. Bergin, Anahita Jalilvand, Valerie Wright, Kathleen L. Wyne, Revati S. Dewal, Lisa A. Baer, Katherine R. Wright, Kristin I. Stanford, Bradley Needleman, Stacy Brethauer, Sabrena Noria, David Renton, Joshua J. Joseph, Amy Lovett-Racke, Joey Liu, Willa A. Hsueh

**Affiliations:** 1 Diabetes and Metabolism Research Center, Division of Endocrinology, Diabetes & Metabolism, Department of Internal Medicine, The Ohio State University Wexner Medical Center, Columbus, OH 43210 USA; 2 Division of Endocrinology, Diabetes & Metabolism, Department of Internal Medicine, Penn State Health Milton S. Hershey Medical Center, Hershey, PA 17033 USA; 3 Diabetes and Metabolism Research Center, Division of Physiology and Cell Biology, Department of Internal Medicine, The Ohio State University Wexner Medical Center, Columbus, OH 43210 USA; 4 Center for Minimally Invasive Surgery, Department of General Surgery, The Ohio State University Wexner Medical Center, Columbus, OH 43210 USA; 5 Department of Microbial Immunity and Infection, The Ohio State University Wexner Medical Center, Columbus, OH 43210 USA

**Keywords:** Translational research, Obesity, Translational immunology, Regulatory T cells

## Abstract

Decreased adipose tissue regulatory T cells contribute to insulin resistance in obese mice, however, little is known about the mechanisms regulating adipose tissue regulatory T cells numbers in humans. Here we obtain adipose tissue from obese and lean volunteers. Regulatory T cell abundance is lower in obese vs. lean visceral and subcutaneous adipose tissue and associates with reduced insulin sensitivity and altered adipocyte metabolic gene expression. Regulatory T cells numbers decline following high-fat diet induction in lean volunteers. We see alteration in major histocompatibility complex II pathway in adipocytes from obese patients and after high fat ingestion, which increases T helper 1 cell numbers and decreases regulatory T cell differentiation. We also observe increased expression of inhibitory co-receptors including programmed cell death protein 1 and OX40 in visceral adipose tissue regulatory T cells from patients with obesity. In human obesity, these global effects of interferon gamma to reduce regulatory T cells and diminish their function appear to instigate adipose inflammation and suppress adipocyte metabolism, leading to insulin resistance.

## Abstract (web summary)

Murine models of obesity suggest that decreases in the adipose tissue regulatory T cell compartment may contribute to insulin resistance, but how this corresponds in the human clinical context is less well understood. Here the authors propose a role for interferon gamma in shrinking the adipose tissue regulatory T cell compartment seen in human obesity and assess the alterations seen during induction of dietary changes.

## Introduction

Obesity is a world-wide pandemic associated with low-grade chronic inflammation^1^. As the major storage depot for excess calories and comprised of a variety of active immune cells, adipose tissue (AT) is uniquely poised at the crossroads of inflammation and metabolism. Therefore, defining the immune cell architecture of AT and mechanisms that lead to enhanced AT inflammation in obesity is critical in combating its metabolic complications. In mice, AT macrophages (ATMs) are the best understood immunomodulators^2^, but elegant studies have identified adipose resident T lymphocytes (ARTs), including pro-inflammatory T helper (Th) type 1 cells, Th2 cells and anti-inflammatory regulatory T cells (Tregs), as important determinants of systemic metabolism^3,4^. In lean mice Tregs protect against inflammation^4,5^, and adoptive transfer of Tregs into obese mice^3,6^ or prevention of the drop in Tregs (13, 15) improves insulin sensitivity.

Despite insights regarding the impact of immune cells in mouse AT, comparatively little is known about human AT, particularly Tregs. Therefore, the goal of this translational investigation was to define AT Treg changes in human obesity and identify potential mechanisms that could explain these changes. Here we show that Tregs are reduced in obese human AT and acutely decline after overfeeding. This AT Treg loss derives from two distinct mechanisms: adipocyte MHCII-mediated adaptive immune activation of Th1 cells (with increased IFNγ production that suppresses Treg differentiation), not previously shown in humans, and excess Treg loss through exhaustion, not previously reported, also robustly impacted by IFNγ. Our comprehensive investigation highlights a central role for IFNγ in mediating obesity-associated inflammation in humans.

## Results

### Decreased Treg abundance in human obese AT associated with insulin resistance

**Fig. 1:**
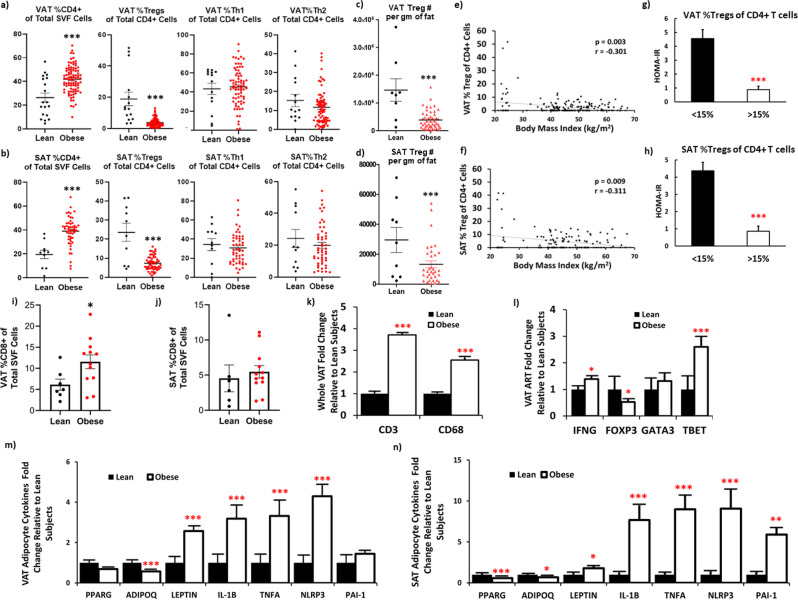
Decreased Treg abundance in adipose tissue (AT) obtained from obese vs. lean subjects associates with insulin resistance. Adipose regulatory T (Treg), Type 1 (Th1), and Type 2 (Th2) helper T lymphocytes as percent of total CD4 + cells in (**a**) visceral (VAT) (*N* = 15 lean independent subjects and *N* = 83 obese independent subjects) and (**b**) subcutaneous (SAT) (*N* = 11 lean independent subjects and *N* = 52 obese independent subjects) AT of individuals analyzed by two-sided *T*-test. AT Tregs per gram of fat tissue in (**c**) VAT (*N* = 50 obese and *N* = 9 lean independent subjects) and (**d**) SAT (*N* = 34 obese and *N* = 9 lean independent subjects). Correlation analyses (Spearman) between %Treg and body mass index (BMI) in (**e**) VAT and (**f**) SAT. Insulin resistance (HOMA-IR) in patients with %Tregs less than (*N* = 73 biologically independent subjects) or greater than 15% (*N* = 5) of total CD4 + T cells in (**g**) VAT and (**h**) SAT, analyzed by two-sided *T*-test. Patients on diabetes medications were excluded from analyses. CD8 + Tcells as a % of SVF in (**i**) VAT (*N* = 7 lean and *N* = 12 obese subjects) and (**j**) SAT (*N* = 6 lean and *N* = 12 obese subjects). (**k**) Whole VAT, (**l**) VAT adipose resident T (ART) cells, (**m**) VAT and (**n**) SAT adipocyte gene expression in lean (black bars) and obese (white bars) subjects by two-sided *T*-test. All means for lean subject gene expression are presented as normalized to 1. Abbreviations: Leptin (*LEP*), Interleukin (*IL*)*−1B*, tumor necrosis factor alpha (*TNF*), NOD-, LRR- and pyrin domain-containing protein 3 (*NLRP3*), plasminogen activator inhibitor-1 (*PAI-1* or *SERPINE1*), adiponectin (*ADIPOQ*), and peroxisome proliferator-activated receptor gamma (*PPARG*). Source data are provided as a Source Data file. Data presented as mean ± SEM. ***p < 0.001; **p < 0.01; *p < 0.05; and ^╤^*p* < 0.20. No replicate samples were utilized.

Tregs as percent (%) of CD4 + cells were substantially decreased in obese VAT (Fig. 1a) and SAT (Fig. 1b). SAT and VAT %Tregs in the same individuals were positively correlated (*r* = 0.790, *p* < 0.001). The number of Tregs per gram of fat tissue was also decreased in obese compared to lean VAT (Fig. 1c) and SAT (Fig. 1d) and correlated with Tregs as % of CD4 + cells (VAT: *r* = +0.412, *p* < 0.001; SAT: *r* = +0.613, *p* < 0.001). In addition, after adjusting for age and gender, significant differences remained between lean subjects and subjects with obesity in %SAT Tregs (*F*(1, 42.7) = 1893, *p* < 0.001) and %VAT Tregs (*F*(1, 56.47) = 2525, *p* < 0.001). VAT and SAT Tregs as %CD4 + cells also correlated with BMI (Fig. 1e, f). However, we found no differences between obese and lean either VAT or SAT %Th1 cells (*p* = 0.719 and *p* = 0.541, respectively) or %Th2 cells (*p* = 0.452 and *p* = 0.727, respectively), although SAT Th1 cells per gram of fat tissue were significantly different between lean subjects and subjects with obesity (1,955,550 ± 689,290 cells/gm of fat vs. 5,959,011 ± 1,006,152 cells/gm of fat, *p* = 0.003) and positively correlated with BMI (*r* = +0.414, *p* = 0.009), and VAT Th1 per gram of fat tissue trended to correlate with HOMA-IR (*r* = +0.306, *p* = 0.055). Representative Treg blots and isotype controls are shown in Supplementary Fig. 1.

We found a marked difference in insulin resistance (HOMA-IR) between those subjects whose AT %Treg composition was greater than or less than 15% (VAT: Fig. 1g; SAT: Fig. 1h). In addition, VAT %Tregs inversely correlated with both insulin resistance (*p* = 0.009) and plasma insulin levels (*p* = 0.007). There were no differences in Tregs, Th1, or Th2 cells in VAT or SAT between subjects with obesity based on the presence or absence of T2D (Supplementary Fig. 2), hypertension, or hyperlipidemia. Although T2D may itself alter the AT Treg population; after excluding subjects with T2D, the %Tregs in obese VAT and SAT remained significantly lower than in lean VAT and SAT (Supplementary Fig. 3). In PB from a smaller number of subjects, we also found decreased Tregs in obese vs. lean individuals (Supplementary Fig. 4), as previously reported^7^. CD8 + T cells as % of SVF were increased in VAT (*p* = 0.036), but not SAT (*p* = 0.619) of obese vs. lean subjects (Fig. 1i, j).

### Adipose resident T cells (ARTs) exhibited lower FOXP3 and higher TBET expression in human obesity

Gene expression of whole fat indicated an increase in T lymphocytes (CD3) and macrophages (CD68) in obese vs. lean VAT (Fig. 1k). *FOXP3* was lower in VAT ARTs of obese vs. lean subjects (Fig. 1l), consistent with the decrease in AT Treg abundance in obesity. However, the CD4 + Th1 marker *TBET* was higher in obese ARTs, which was not found in flow analyses, possibly because Th1 cells were not activated prior to flow. *IFNG* expression was also increased in obese ARTs. There was no difference in ARTs expression of *GATA3*, a marker of CD4 + Th2 cells. These changes in expression of ARTs genes resemble our findings in mice^8,9^.

### Adipocytes from individuals with obesity expressed increased pro-inflammatory adipokines that did not correlate with AT Treg abundance

In obese vs. lean VAT and SAT, we found increased adipocyte gene expression of pro-inflammatory adipokines (*LEP*, *IL-1B*, *TNFA*, *NLRP3*) and lower gene expression of the anti-inflammatory factors *ADIPOQ* and *PPARG* (Fig. 1m, n, respectively), consistent with prior reports^10–13^. VAT *LEP* and SAT *TNFA*, *NLRP3*, and *LEP* were significantly related to HOMA-IR, but after adjusting for BMI, none remained predictive of HOMA-IR or fasting insulin. Of all the cytokines/adipokines measured, we found a direct association only between VAT Tregs and adipocyte *ADIPOQ* expression (Supplementary Fig. 5).

### Obese adipocytes demonstrated increased expression of MHCII genes and can present antigen to activate CD4 + T cells and augment IFNγ production

**Fig. 2:**
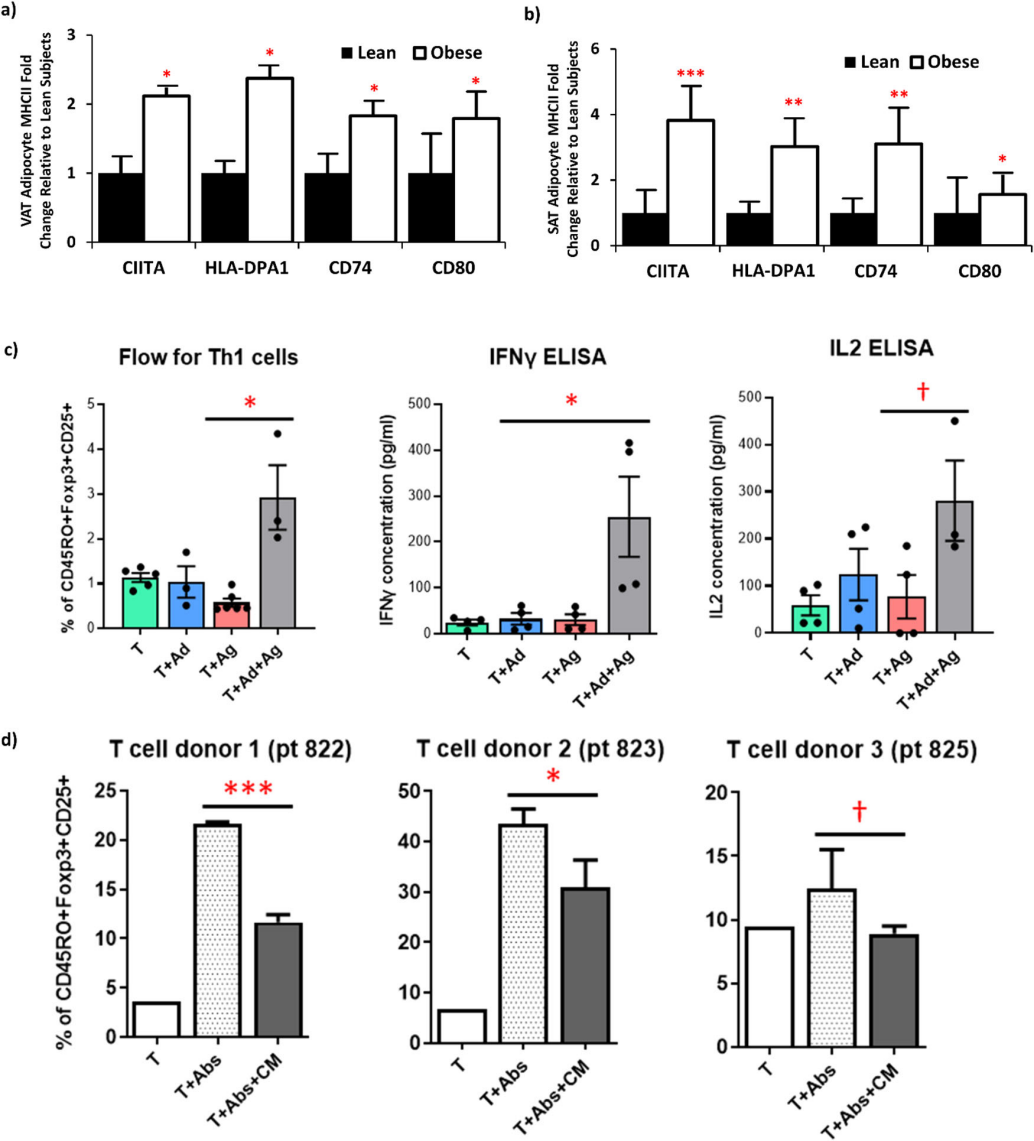
Human adipocyte antigen presentation contributes to adipose tissue inflammation. **a** Visceral (VAT) and **b** Subcutaneous (SAT) adipocyte gene expression in lean (*N* = 15) and obese (*N* = 83) subjects of MHCII-related genes by two-sided independent sample *T*-test. Source data are provided as a Source Data file. Means for lean subject gene expression are presented as normalized to 1. **c** Co-culture of CD4 + T cells from human obese subjects in the presence/absence of adipocytes and/or antigen (Copaxone (50 µg/ml)) assessing the % Th1 cells of total CD4 + cells by flow cytometry and IFNγ and IL-2 media levels by ELISA analyzed by student *T* test (without adjustment for multiple comparisons). **d** Treg differentiation assay: naïve CD4 + T cells (T) from new donors co-cultured, with or without CD3/CD28 antibodies (Abs) to activated T cells, and with conditioned media (CM: IFNγ and IL-2 high) from the previous experiments in c to determine %Tregs of CD4 + cells analyzed by student *T* test (without correction for multiple comparisons). See text for abbreviations. Data presented as mean ± SEM. ***p < 0.001; **p < 0.01; *p < 0.05; and ^†^*p* < 0.10. No replicate samples were utilized.

Adipocytes from humans with obesity had increased expression of multiple MHCII-related genes (*CIITA*, *HLA-DPA1*, *CD74*, *CD80*) in both VAT (Fig. 2a) and SAT (Fig. 2b). These findings are similar to those in obese mice^8,9^. Associations between MHCII genes and BMI are listed in Supplementary Table 3, indicating a strong relationship between higher adipocyte MHCII antigen presenting capability and increasing adiposity. Moreover, adipocyte VAT *CIITA* and SAT *CD74* expression were negatively associated with Treg abundance (Supplementary Fig. 5). However, we found no significant associations between any MHCII-related genes and VAT or SAT Th1 or Th2 cell abundance.

To determine the functional significance of enhanced adipocyte MHCII expression, we co-cultured isolated adipocytes with bead-isolated CD4 + T cells obtained from PB of the same subjects with obesity (*n* = 3) using Copaxone as the antigen, which has been shown to enhance CD4 + Th1 cell differentiation in multiple sclerosis^14^. Culture of PB naïve T cells with adipocytes alone or with antigen alone did not alter the abundance of CD4 + Th1 cells, measured by flow cytometry, or IFNγ or IL-2 levels in the co-culture media (Fig. 2c). However, when antigen was added, there was a 3-fold increase in CD4 + Th1 cells, accompanied by a ~10-fold increase in media levels of both IFNγ and IL-2. To determine whether this increase in Th1 cells could impact Tregs, the supernatant of these co-cultures was added to naïve PB CD4 + T cells incubated under Treg differentiation conditions. Accordingly, the supernatants impaired Treg differentiation in naïve T cells obtained from blood of two different donors, with a trend in a third donor (Fig. 2d).

### Regulatory T cells (Tregs) decreased acutely following a hypercaloric, high-fat diet (HFD) and were related to insulin sensitivity

**Fig. 3:**
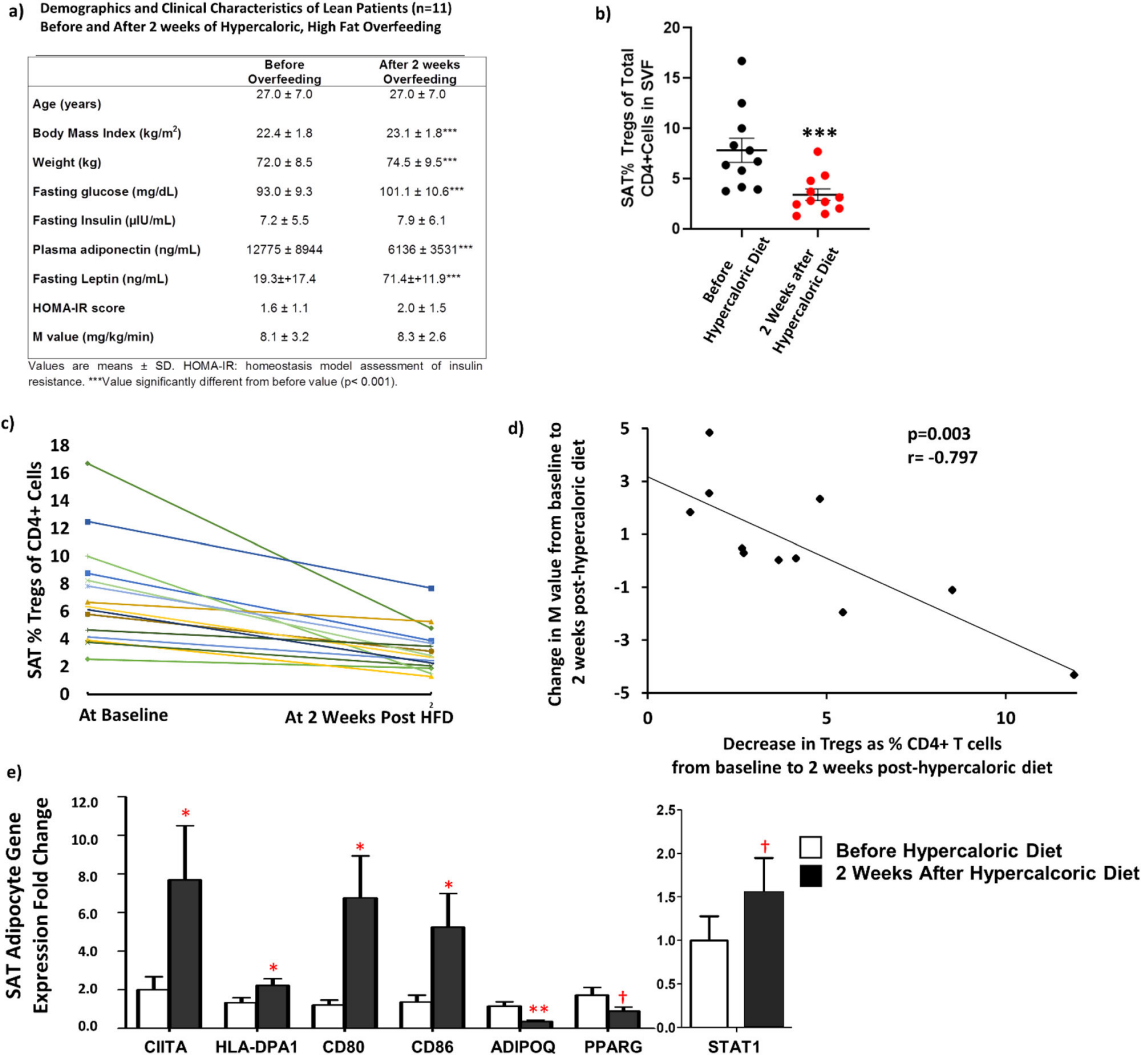
SAT Tregs decrease acutely following a hypercaloric, high-fat diet and are related to adipocyte MHCII gene expression and insulin resistance. **a** Clinical Characteristics of lean subjects (*N* = 11) before and after 2 weeks of a hypercaloric, high saturated fat diet (HFD). Change in SAT Tregs as % of total CD4 + cells in the stromal vascular fraction (SVF) from before to after 2 weeks HFD (*N* = 11) by Wilcoxon signed ranks test (**b**) as a group and (**c**) in each individual participant. **d** Correlation (Spearman) between the change in M value (*N* = 11) and decrease in SAT Tregs as percent (%) of total CD4 + cells in the SVF from before to after 2 weeks post HFD. Correlations were two-sided without correction for multiple comparisons. **e** SAT adipocyte gene expression of MHCII-related genes, STAT1, and adipokines/cytokines from before to after 2 weeks post HFD. See text for abbreviations. Data were analyzed by two-sided independent sample *T*-test (without adjustment for multiple comparisons). Source data are provided as a Source Data file. Data presented as mean ± SEM. ***p < 0.001; **p < 0.01; *p < 0.05; and ^†^*p* < 0.10. No replicate samples were utilized.

We obtained SAT samples from healthy, lean volunteers before and after 2 weeks of HFD consumption. The subjects had a small but significant increase in BMI, body weight, fasting plasma glucose and leptin, and a drop in adiponectin, but no detectable changes in fasting insulin, HOMA-IR, or M value by hyperinsulinemic-euglycemic clamp (Fig. 3a).

However, SAT %Tregs consistently decreased from before to after 2 weeks of overfeeding (Fig. 3b), with no changes in %Th1 cells (*p* = 0.361) or %Th2 cells (*p* = 0.937). Each individual participant experienced a decrease in SAT Tregs at 2 weeks HFD (Fig. 3c). The drop correlated with the change in overall insulin sensitivity by clamp (M value, Fig. 3d), such that the greater the reduction in Tregs, the greater the decrease in insulin sensitivity. This relationship persisted despite adjusting for, age, gender, pre-intervention BMI, and change in weight from pre to post intervention (*p* = 0.012). In SAT adipocytes, there was a decrease in *ADIPOQ* and *PPARG* and increases in several MHCII-related genes (Fig. 3e). In addition, expression of *STAT1*, which mediates IFNγ and other cytokine signaling, was increased (Fig. 3e). Taken together, these results suggest that increased IFNγ signaling and loss of anti-inflammatory AT Tregs occurs early during chronic HFD ingestion.

### Human obesity was associated with a decline in adipocyte fatty acid flux and mitochondrial function genes that related to Treg abundance

**Fig. 4:**
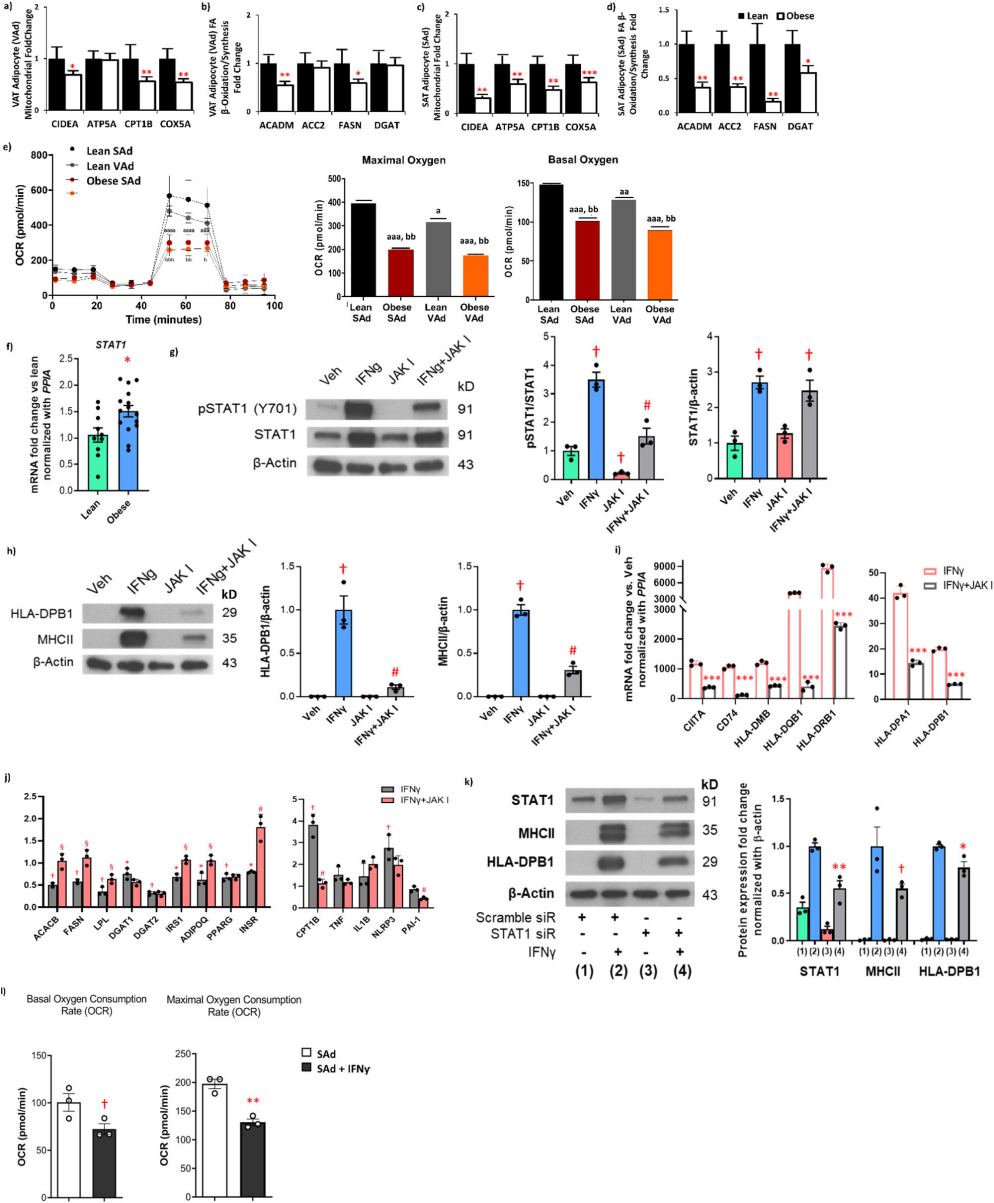
Adipocyte mitochondrial function and fatty acid flux genes decrease in subjects with obesity and relate to AT Treg abundance and IFNγ-enhanced MHCII expression. VAT adipocyte (VAd) and SAT adipocyte (SAd) gene expression in lean (*N* = 15) and obese (*N* = 83) subjects of markers of mitochondrial function (**a**, **c**) and fatty acid oxidation/synthesis (**b**, **d**); ***p < 0.001; **p < 0.01; *p < 0.05 analyzed by two-sided, independent *T* test. **e** Maximal and basal oxygen consumption rate (OCR) in lean (*N* = 2) and obese (*N* = 3) human SAd and VAd by Seahorse; ^a^*P* < 0.05, ^aa^*P* < 0.01, and ^aaa^*P* < 0.001 vs. lean SAd; ^b^*P* < 0.05, ^bb^*P* < 0.01, and ^bbb^*P* < 0.001 vs. lean VAd. **f** VAd gene expession of STAT1 in lean (*N* = 11) and obese (*N* = 15) subjects (*P < 0.05) analyzed by two-sided, independent *T* test without correction for multiple variables. Protein levels of (**g**) STAT1 and (**h**) MHCII-related genes in mature SAd treated with a JAK inhibitor (JAK I, 10 µM) with or without recombinant IFNγ (20 ng/ml) for 48 hr; ^†^*P* < 0.01 vs. Vehicle; ^#^*P* < 0.01 vs. IFNγ (*N* = 3). Data was analyzed by two-sided, independent *T* tests without correction for multiple variables. **i** Gene expression of MHCII-related genes in mature SAd treated with a JAK inhibitor (JAK I, 10 µM) with or without IFNγ (20 ng/ml) for 48 hr; ***P < 0.001 vs. IFNγ (*N* = 3). **j** Gene Expression of fatty acid oxidation/synthesis and adipokines/cytokines in adipocytes treated with a JAK inhibitor (JAK I, 10 µM) with or without IFNγ (20 ng/ml) for 48 hr. *P < 0.05 and ^†^*P* < 0.01 vs. Vehicle (Its mean is 1 and bars not shown); ^§^*P* < 0.05 and ^#^*P* < 0.01 vs. IFNγ (*N* = 3). **k** In differentiated SAd, STAT1 siRNA decreased STAT1 protein and attenuated the IFNγ (20 ng/ml) effect to stimulate MHCII proteins, while scrambled siRNA had no effect. *P < 0.05 and **P < 0.01, and ^†^*P* < 0.10 vs. scramble siRNA+IFNγ (*N* = 3). **l** Maximal and basal OCR from human SAd + /- IFNγ (*N* = 3). All data presented as mean ± SEM. Source data are provided as a Source Data file.

Obese VAT adipocytes had diminished expression of several mitochondrial-related genes (*CIDEA, CPT1B*, *COX5A*), and genes involved in fatty acid β-oxidation/synthesis (*ACADM* and *FASN*) (Fig. 4a, b). Similarly, adipocytes from obese SAT also showed decreased expression of genes involved in mitochondrial function (*CIDEA*, *ATP5A*, *CPT1B*, and *COX5A*) and fatty acid β-oxidation/synthesis (*ACADM*, *ACC2*, *FASN*, *DGAT*) (Fig. 4c, d). Obese VAT adipocytes also revealed an increase in STAT1 expression (Fig. 4f). Significant inverse associations between mitochondria and fatty acid-related genes with BMI are listed in Supplementary Table 3 and indicate that with increasing BMI, there is a corresponding decline in adipocyte metabolic function. Seahorse analyses was consistent with the gene expression data, demonstrating a marked decrease in both basal and maximal AT oxygen consumption rate (OCR) (Fig. 4e) and non-mitochondrial respiration (Supplementary Fig. 6) in obese human VAT and SAT adipocytes.

In VAT, we found positive associations between %Tregs and *ACADM* and *CPT1B*. In SAT, significant associations with %Tregs were observed for *ACADM*, *ATP5A*, and *CPT1B* (Supplementary Fig. 5). In VAT and SAT, MHCII-related genes inversely correlated with markers of mitochondrial function and fatty acid β-oxidation/synthesis (Supplementary Table 3). Multiple inverse associations also remained significant after multivariate adjustment for BMI, age, and gender: (1) VAT *CD74* with *CIDEA*, *ATP5A*, and *CPT1B*, (2) VAT *CIITA* with *CIDEA*, *ATP5A* and *CPT1B*, (3) SAT *CIITA* with *ATP5A*, *CPT1B*, *CIDEA* and *ACC2*, and 4) SAT *CD80* with *ATP5A*, *CPT1B*, *CIDEA*, *ACC2* and *ACADM*. Even when lean subjects were excluded, multiple relationships remained between expression of MHCII pathway components and metabolic genes (Supplementary Table 4), indicating a potential common factor, IFNγ, both increases the MHCII pathway and suppresses the metabolic pathway in adipocytes. Taken together, these data indicate that adipocyte metabolic function becomes impaired with progressive obesity, while adaptive immune capability increases, underscoring the likely dual effects of IFNγ.

### Interferon gamma alters adipocyte metabolism through the JAK/STAT1 pathway

Given our finding that STAT1 adipocyte gene expression was higher in human obesity (Fig. 4f) and increases after overfeeding (Fig. 3e), and that IFNγ-mediated increases in STAT1 can impair Treg differentiation^15^, we next examined the effects of IFNγ and its signaling through the JAK/STAT1 pathway on metabolic genes. IFNγ prominently increased STAT1 phosphorylation (Fig. 4g) in cultured human SAT adipocytes, an effect that was attenuated by a JAK inhibitor. However, there was no or little IFNγ-mediated effect on STAT3 or STAT5 (Supplementary Fig. 7). IFNγ markedly increased MHCII protein levels and gene expression, also attenuated by a JAK inhibitor (Fig. 4h, i). In sharp contrast, IFNγ diminished expression of several genes involved in fatty acid synthesis and metabolism (*FASN*, *LPL*, *ACC2*, *DGAT1*, *DGAT2*), as well as insulin receptor substrate 1, *IRS1*, and the insulin receptor, *INSR* (Fig. 4j); most of these effects were reversed by JAK inhibition. Although IFNγ increased *CPT-1B* expression, it did not have a marked effect on most mitochondrial genes. IFNγ also suppressed expression of *ADIPOQ* and *PPARG*, and enhanced *TNF* and *NLRP3*, but not *IL1B* or *PAI-1* (Fig. 4j). To confirm the role of STAT1, we added IFNγ in the presence of a STAT1 siRNA or scrambled siRNA (Fig. 4k). The STAT1 siRNA, but not scrambled siRNA, decreased STAT1 protein and impaired IFNγ stimulation of MHCII and HLA-DPB1, indicating that the IFNγ effects were mediated by STAT1. Maximal and basal oxygen consumption rate (OCR) in cultured human adipocytes from human SAT were also blunted by IFNγ (Fig. 4l).

### Interferon gamma contributed to exhaustion in human Tregs

**Fig. 5:**
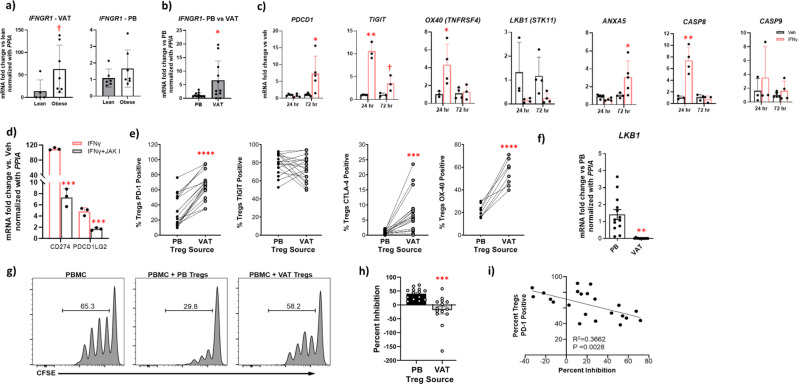
Interferon gamma mediates an exhausted human regulatory T cell phenotype. **a** Gene expression of interferon gamma receptor 1 (*IFNGR1*) in isolated human regulatory T cells (Tregs) from visceral adipose tissue (VAT) and peripheral blood (PB) of lean versus obese subjects (VAT: *N* = 5 lean, *N* = 7 obese; PB: *N* = 6 lean, *N* = 7 obese). **b** Gene expression of *IFNGR1* in isolated human Tregs from VAT vs. PB. **c** Gene expression of programmed cell death protein 1 (*PDCD1* or *PD-1*), T cell immunoreceptor with Ig and ITIM domains (*TIGIT*), *OX40*, *LKB1*, Annexin 5 (*ANXA5*), and Caspase (*CASP*) *8* and *9* in isolated human PB Tregs (*N* = 4 per group) cultured for 24 hr and 72 hr, with or without recombinant IFNγ (20 ng/ml); **P < 0.010; *P < 0.05. **d** Gene expression in human cultured subcutaneous adipocytes of the PD-1 ligands *PD1GL1* (*CD274*) and *PDCD1LG2*. Adipocytes were pretreated with a JAK inhibitor (JAK I) for 1.5 hr in adipocyte maintenance medium prior to co-treatment with IFNγ (20 ng/ml) for 48 hr (*n* = 3). Fold changes vs. vehicle samples are not shown in the graph and all close to 1; ***P < 0.001. **e** VAT Tregs and PB Tregs in lean (*N* = 6) and obese (*N* = 12) human subjects positive for PD-1, TIGIT, and CTLA4 and in lean (*N* = 1) and obese (*N* = 7) human subjects positive for OX-40 by flow cytometry; ****P < 0.0001; ***P < 0.001; **P < 0.010. **f** Gene expression of *LKB1* in human VAT (*N* = 13) vs. PB (*N* = 13) from the same individuals; **P < 0.010. **g** % proliferation in cultured activated peripheral blood mononuclear cells (PBMCs) without Tregs (first panel) and with flow sorted Tregs from PB (second panel) and VAT (third panel) in a representative obese patient. **h** % inhibition of proliferation in cultured activated peripheral blood mononuclear cells (PBMCs) with Tregs flow sorted from PB (*N* = 14) vs. VAT (*N* = 14) from the same individuals, ***P < 0.001. **i** Correlation between % of total Tregs positive for PD-1 vs % inhibition of proliferat**i**on in cultured PBMCs with flow sorted Tregs from VAT and PB. Source data are provided as a Source Data file. Group comparison data was analyzed by two-sided, independent *T* tests without correction for multiple variables and presented as mean ± SEM.

We found that the IFNG receptor 1 (*IFNGR1*) was more highly expressed in obese vs lean human VAT Tregs (Fig. 5a) with no differences in PB Tregs. *IFNGR1* expression was also substantially higher in VAT compared to PB Tregs (Fig. 5b). We therefore examined the effect of IFNγ in cultured human Tregs on regulating expression of markers of apoptosis and key inhibitory co-receptors, important rheostats of T cell function^16^. Recombinant IFNγ (2 ng/ml) treatment of isolated human PB Tregs demonstrated increased expression of co-receptors *PD-1*, *TIGIT*, and *OX40*, and decreased *LKB1* expression (a key Treg supportive factor^17^) (Fig. 5c). We also noted higher annexin 5 and caspases 8 and 9 (markers of cell apoptosis) (Fig. 5c). IFNγ enhanced both PD-1 ligand 1 (*PDL-1*; *CD274*) and 2 (*PDL-2; PDCD1LG2)* expression in human adipocytes, which was reversed by JAK inhibition (Fig. 5d). Congruent with gene expression, flow analyses revealed increased PD-1, OX40, and CTLA4 expression in VAT vs PB Tregs isolated from the same obese individuals, although TIGIT was not increased (Fig. 5e). In addition, LKB1 expression was markedly decreased in VAT vs. PB Tregs (Fig. 5f).

Because VAT Tregs had a greater proportion of PD-1, OX40, and CTLA4 positive cells compared to PB, we cultured activated PBMCs with Tregs flow sorted from VAT and PB of the same human subjects to compare their function (suppression of proliferation). Compared to PBMCs cultured without Tregs, PBMCs cultured with PB Tregs inhibited proliferation by 30% compared to *no* inhibition of proliferation by VAT Tregs, *p* < 0.001 (Fig. 5g, h). Notably, levels of PD-1 positive Tregs in cultures had an inverse correlation with inhibitory capacity (Fig. 5i). Thus, in human VAT, the function of Tregs is reduced in association with higher PD-1, a potential marker of Treg exhaustion.

## Discussion

**Fig. 6:**
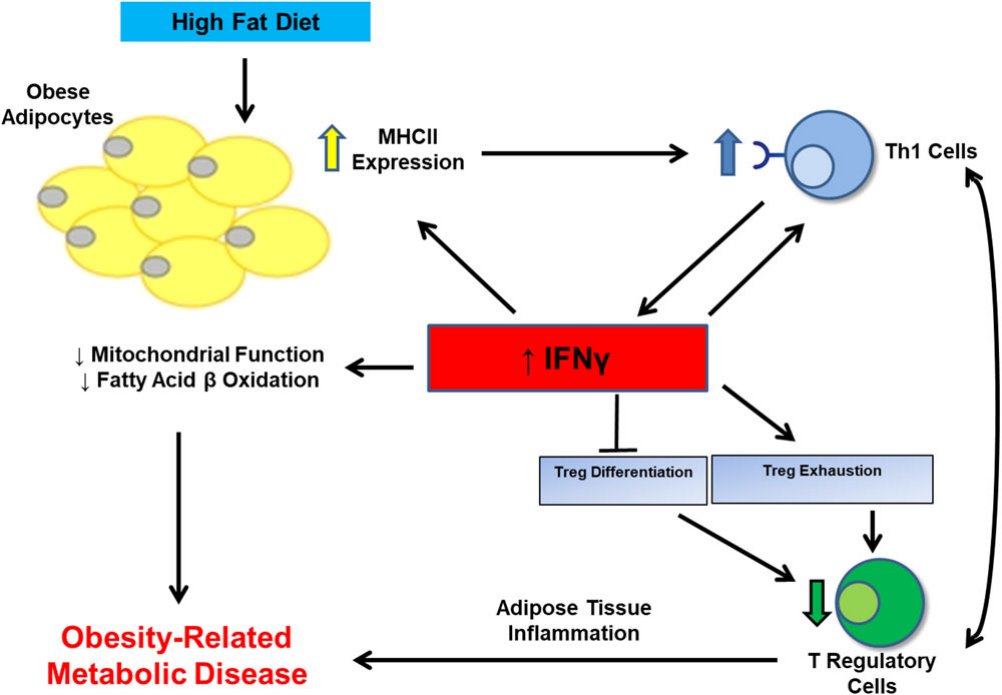
Summary of the proposed mechanism for adipocyte-mediated insulin resistance in human obesity, illustrating the central role of interferon gamma. Obese adipocytes acquire increased antigen presenting capability leading to an increase in CD4 + Th1 cells in AT that produce IFNγ. Increased IFNγ suppresses Treg differentiation and enhances Treg exhaustion, augments adipocyte MHCII pathway which is not common to other cytokines, and attenuates adipocyte mitochondrial function and fatty acid flux to create an escalating cycle of inflammation and insulin resistance. Exhaustion features in human Tregs include increased expression of inhibitory co-receptors and markers of apoptosis and suppressed LKB1.

Our comprehensive investigation of Tregs in obese vs. lean human AT indicates that both VAT and SAT Treg abundance decrease in obesity and early during ingestion of HFD. This decline is associated with systemic insulin resistance. Leptin, which is increased after two weeks HFD, can increase CD4 + Th1 cell differentiation^18^, leading to an early increase in IFNγ. Our finding of increased adipocyte expression of STAT1, a major IFNγ intracellular mediator^19^ that may also be activated by other inflammatory factors^20,21^, in both chronic obesity and after 2 weeks HFD suggests increased IFNγ activity and inflammation under these conditions. Elevated IFNγ levels are further perpetuated by an escalating cycle of inflammation in which Th1 cell activation leads to more IFNγ that enhances adipocyte MHCII pathway activation to further increase Th1 cells. Moreover, IFNγ also has profound Treg effects to impair differentiation, as we have previously shown in mice^9^, and to promote exhaustion. Therefore, AT inflammation is initiated and perpetuated, at least in part, from the global actions of IFNγ (Fig. 6).

Relative to mice, very little is known about AT T cell changes in human obesity^22^. Four studies found decreased *FOXP3* gene expression in obese vs. lean VAT^6,23–25^, but a fifth reported the opposite^26^. Only three studies have performed human AT T cell flow analyses^3,27,28^. McLaughlin et al.^27^ found that Th1 cells correlated directly and Th2 cells inversely with plasma C-reactive protein concentrations in 10 overweight/obese subjects, suggesting that AT T cells contribute to systemic inflammation. However, they did not assess Tregs or include lean subjects. Gyllenhammer et al.^28^ measured Tregs in SAT and VAT of 44 subjects with obesity and found a negative association with fasting glucose; lean subjects were also not studied. More recently, Vijay et al.^29^ performed single cell RNAseq analyses of human SVF but did not detect Treg changes. Our results comparing larger numbers of both lean individuals with those with obesity demonstrate that Treg abundance falls by >70%, from ~20% of CD4 + cells in lean to ~5% in obesity in both VAT and SAT (Fig. 1). In AT of lean mice Tregs comprise 50% of the CD4 + T cells, decreasing to less than 20% in obesity^4^. This decline appears to be a key contributor to obesity-induced insulin resistance as suggested by our observations herein demonstrating that human adipocytes can present antigen to activate IFNγ-producing Th1 cells and that ingestion of HFD induces a rapid drop in AT Tregs.

Human adipocytes cultured with T cells have previously been shown to stimulate proliferation in a contact dependent manner associated with increased expression of MHCII pathway genes; however, the investigators did not directly demonstrate adipocyte antigen presentation^30^. In the current investigation, we show that human adipocytes can present antigen. In the presence of copaxone, obese human adipocytes co-cultured with naïve PB T cells increased Th1 cell differentiation, as well as secretion of IFNγ and IL-2, a major T cell growth factor (Fig. 2). We further demonstrate that the supernatant (containing high amounts of IFNγ and IL-2) from these co-cultures inhibited Treg differentiation, suggesting Th1 cells inhibit induction of human Tregs through production of IFNγ as previously shown in mice^31^.

In healthy, lean volunteers, HFD induced a decline in AT Treg abundance after only 2 weeks, before significant weight gain (Fig. 3), paralleling the time course in mice^8^. After 4 weeks of overfeeding, Tam et al. demonstrated that subjects became insulin resistant after minimal weight gain, with no change in SAT CD68 and MCP-1 or SVF CD11b expression, suggesting no major ATM changes^32^. Similarly, in mice, we did not see ATM changes within 2-4 weeks of HFD^8^. In contrast, AT Tregs dropped in *all* 11 individuals administered HFD for 2 weeks, while adipocyte MHCII pathway gene expression also increased. Thus, we believe that adipocyte/T cell crosstalk is an important instigator of AT inflammation in both humans and mice.

In our subjects with obesity, adipocyte MHCII activity increased in association with a widespread reduction in adipocyte genes involved in mitochondrial function and fatty acid oxidation/synthesis (Supplementary Table 2), supported by decreased mitochondrial oxygen consumption rates (Fig. 4). In obese mice, however, we did not see a consistent loss of adipocyte metabolic function^33^. IFNγ through enhanced STAT1 expression and phosphorylation mediates this profound phenotypic switch in human adipocytes, inducing MHCII genes, while suppressing metabolic genes and adiponectin. Moreover, the footprint of excess IFNγ is suggested by increased adipocyte STAT1 expression in obesity and following short-term HFD administration in lean subjects (Fig. 3). These metabolic findings resemble investigations in human Simpson-Golabi-Behmel syndrome adipocytes (62) (in which IFNγ decreased insulin-mediated glucose uptake, also reversed by a JAK1 inhibitor), 3T3-L1 adipocytes^34^, and primary adipocytes from obese individuals^35^, but extend these findings. These changes in adipocyte function are implicated as important contributors to systemic insulin resistance (summarized in^36^). The dual effects of IFNγ likely explain the inverse relationships of MHCII pathway genes and metabolic pathway genes in the adipocyte in obesity.

Finally, our studies indicate a role for IFNγ in human Treg apoptosis and death in obesity through a process resembling exhaustion. CD8 + T cell exhaustion, well characterized in states of chronic infection (HIV, Hepatitis, etc.) and malignancy, is defined by poor effector function, decreased IL-2 production, and overexpression of inhibitory co-receptors^37–39^. In particular, the PD-1 pathway has a major impact on survival and proliferation of exhausted CD8 + T cells^40,41^. PD-1 has also been implicated in Treg exhaustion in cancer related to increased IFNγ and autoimmune disease in humans^42,43^, and in conjunction with attenuated LKB1 expression in mice and humans^44^.

We found that IFNγ increased expression of *PD-1*, *TIGIT*, *OX40* and apoptosis markers and suppressed *LKB1* in cultured human Tregs (Fig. 5), and robustly stimulated the PD-1 ligand in cultured human adipocytes. Flow analyses revealed a 2-fold increase in PD-1 positive Tregs, a 3-fold increase in OX40 positive Tregs, and an 8-fold increase in CTLA4 positive Tregs in VAT compared to blood, as well as a nearly 15-fold decrease in LKB1 expression. Furthermore, blood Tregs inhibited PBMC proliferation by 30% compared to no suppression by VAT Tregs, and proliferation correlated with the number of PD-1 positive cells in co-culture. Thus, Tregs in obese human AT are highly dysfunctional, likely due to exhaustion. Recently, Porsche, et al.^45^ published that all VAT T cells are exhausted in obesity. Cultured SVF cells were treated with αCD3/CD28 Dynabeads, and both VAT CD8 + and CD4 + Teff cells from obese mice increased CD25 + cells less in response to activation compared to lean mice. However, CD25 is constitutively expressed on Tregs, so activation could not be appropriately measured in Tregs. Our results provide evidence that AT Tregs may undergo an exhaustive process in the setting of human obesity. Further studies are necessary to definitively characterize exhausted human AT Tregs.

In conclusion, the results from our translational study offer new insights into human AT Tregs, demonstrating striking differences in Tregs in lean vs. obese human AT and an acute decline in Tregs after overfeeding. This AT Treg loss appears to be a consequence of two distinct mechanisms: adipocyte adaptive immune activation of Th1 cells with increased IFNγ production that suppresses Treg differentiation, and excess Treg loss through exhaustion, also robustly impacted by IFNγ. These results in humans could lead to new avenues of investigation and immune-based therapies for obesity.

## Methods

Our research complies with all relevant ethical regulations. The study was approved by The OSU Institutional Review Board (IRB), and all participants provided written informed consent. Although human subjects were involved in the study, they were not prospectively assigned to an intervention to evaluate the effect on health-related biological outcomes and the intervention was not comparing diets or treatments. Therefore, the study is exempt from registration in Clinical Trials.gov.

### Surgical study population

VAT and SAT biopsies and peripheral blood samples were obtained from obese (*n* = 83, age 45.0 ± 11.3 yo, BMI 48.8 ± 8.5 kg/m^2^) and lean (*n* = 15, age 42.9 ± 12.3 yo, BMI 23.6 ± 1.8 kg/m^2^) patients who underwent elective surgery at The Ohio State University (OSU) Center for Minimally Invasive Surgery (Table 1). Subjects received no compensation for participation. Elective surgery in the lean subjects consisted of 6 cholecystectomies, 6 hernia repairs, 2 Nissen fundoplications, and 1 myotomy. Elective bariatric surgery in the obese group consisted of 47 subjects undergoing sleeve gastrectomy and 36 undergoing Roux-en-Y gastric bypass. 0/15 (0%) of the lean subjects and 20/83 (24.1%) of the obese subjects had Type 2 Diabetes Mellitus.

**Table 1:** Demographics and Clinical Characteristics of Lean and Obese Patients

	Lean subjects (*n* = 15)	Obese subjects (*n* = 83)
#Female/#Male (% Female)	7/8 (47)	58/25 (70)
Age (years)	42.9 ± 12.3	45.0 ± 11.3
BMI (kg/m^2^)	23.6 ± 1.8	48.8 ± 8.5***
Fasting glucose (mg/dL)	82.8 ± 14.5	90.3 ± 21.7*
Fasting Insulin (µIU/mL)	5.0 (1.6–6.9)	14.5 (8.1–26.3)**
HOMA-IR	1.0 (0.3–1.7)	3.1 (1.5–6.3)*
Plasma adiponectin (ng/mL)	9772 (5540–20851)	5451 (3347–8752)***
Plasma Leptin	27.8 ± 10.3	51.8 ± 43.5***
Systolic Blood Pressure (mmHg)	124.4 ± 22.4	133.0 ± 17.1
Diastolic Blood Pressure (mmHg)	70.4 ± 10.3	75.8 ± 8.2
Total Cholesterol (mg/dL)	185.0 ± 25.3	176.6 ± 34.2
LDL Cholesterol (mg/dL)	101.6 ± 35.7	93.4 ± 27.7
HDL Cholesterol (mg/dL)	57.5 (45.3–76.8)	45.5 (38.0–53.5)
Triglycerides (mg/dL)	117.3 ± 105.9	167.3 ± 76.1
AST	64.7 ± 155.6	27.2 ± 21.3
ALT	31.5 ± 9.3	27.9 ± 19.0
# Subjects on ACE inhibitor or ARB (%)	0/15 (0)	39/83 (47.0)
# Subjects on Statins (%)	0/15 (0)	33/83 (39.8)
# Subjects with Type 2 Diabetes Mellitus (%)	0/15 (0)	20/83 (24.1)
# Subjects with Hypertension (%)	4/15 (26.7)	51/83 (61.4)
# Subjects with Hyperlipidemia (%)	0/15 (0)	37/83 (44.6)

Values are means ± SD or median (interquartile range) for non-normalized variables. HOMA-IR: homeostasis model assessment of insulin resistance. Subjects using insulin, sulfonylureas, and/or metformin were excluded from analyses of HOMA-IR. Value significantly different from lean value, ***p < 0.001; **p < 0.01; and *p < 0.05. Source data are provided as a Source Data file.

### Exclusion criteria

For surgical patients exclusions included: current smokers, evidence of end-stage renal or liver disease, history of prior organ transplantation, on chronic pharmacologic steroid or anti-inflammatory use, history of neoplastic disease or chemotherapy within the prior year, Acquired Immune Deficiency Syndrome or immunocompromised, greater than (>) 10% body weight loss within 3 months of enrollment.

### Weight gain protocol

A separate group of lean metabolically healthy patients (*n* = 11, 9 males and 2 females, age 27.0 ± 7.0 yo, baseline BMI 22.4 ± 1.8 kg/m^2^) were instructed by a registered dietician (RD) to consume at minimum an additional 1320 kcal/day with >50% of total caloric intake in total fat and >10% in saturated fats by dining at fast-food restaurants (using meal vouchers) combined with a high calorie liquid formula, TwoCal® HN. TwoCal® HN is a nutritionally complete formula that provides 475 calories per 8 fluid ounces. Subjects were instructed not to change their level of physical activity or start any new medications during the study. The study was approved by The OSU Institutional Review Board (IRB), and all participants provided written informed consent. Subjects were compensated for participation. All study team members were certified by the Collaborative Institutional Training Initiative (CITI Program) throughout the study period, including training in Human Subjects Research and Good Clinical Practice. Exclusion criteria for those participating in the overfeeding study included: Fasting triglycerides >150 mg/dL or nonfasting triglycerides >250 mg/dL, LDL-Cholesterol level >150 mg/dL, presence of significant anemia (hemoglobin < 10.0 gm/dL), current or planned pharmacologic treatment during the study period with blood thinners, aspartate aminotransferase (AST) or alanine aminotransferase (ALT) above the upper limit of normal in the previous year, current smokers, end-stage renal or liver disease, prior organ transplantation, pharmacologic steroid or anti-inflammatory use for >6 months in the last 6 months, neoplastic disease or chemotherapy within the prior year, Acquired Immune Deficiency Syndrome, >10% body weight loss within 3 months of enrollment, or positive urine pregnancy test.

The subjects were followed closely by the RD, who advised subjects on what to eat and how to incorporate TwoCal® HN supplements into their individualized meal plans, in order to match the rate of weight gain. After completion of the weight gain protocol, each participant met individually with the RD to plan a hypocaloric diet to facilitate losing the weight gained, if desired.

After obtaining informed consent, subjects were screened with a detailed history, including a review of medical records. Patient demographic data including height, weight, BMI, waist to hip ratio, age, gender, race, ethnicity, medications, and medical conditions were collected. Routine blood tests were performed including measurement of hemoglobin A1c (if not done in the last 3 months), fasting glucose and insulin, complete blood counts (CBC), electrolytes, 25 OH vitamin D, thyroid stimulating hormone, liver function tests, lipid panel, serum markers of inflammation, and women of child-bearing potential were required to have a urine pregnancy test. Participants with a positive urine pregnancy test were excluded from the study. The participants filled out a 3-day food record prior to each subsequent meeting with the RD. This 3-day food record required the patient to record everything they eat and drink over a 3 day period on a standardized, validated form. Abdominal SAT was obtained during a 4 h *hyperinsulinemic-euglycemic clamp* procedure (7) at baseline and after 2 weeks of the weight gain protocol. Under sterile conditions, SAT was aspirated with a liposuction cannula with local anesthesia (lidocaine 1% with 1:100,000 epinephrine) and immediately taken to the laboratory on ice.

### Hyperinsulinemic-euglycemic clamp procedure

Intravenous catheters were inserted into an antecubital vein of one arm for insulin infusion and a second catheter was inserted into a hand vein which was heated to obtain “arterialized” blood samples. The clamp was continued for a total of 4 h. At the start of the clamp, insulin was infused at a priming rate of 200 mU.m2.min-1 for 5 min., then decreased to 100 mU.m2.min-1 for 5 min, then decreased to 50 mU.m2.min-1 for the remainder of the 3 h study. This rate was chosen as the minimal rate of insulin to maximally stimulate glucose uptake into skeletal muscle in previous studies. During the insulin infusion, blood samples were taken every 10 min to help determine titration of a 20% dextrose infusion. As insulin lowers blood sugar levels, 20% dextrose was infused in order to achieve euglycemia (glucose ~100 mg/dL) by the end of the 4 h. The rate of the 20% dextrose infusion was titrated every 10 min. Insulin sensitivity was defined by the glucose infusion rate (GIR), the rate of 20% dextrose infusion at the end of the 3 h needed to maintain euglycemia i.e. the higher the GIR the higher the insulin sensitivity, and the M value defined as (GIR*184/60)/patient weight^46^. Blood samples were taken at 7 min, 14 min and 20 min during the last 20 min of the insulin clamp to determine plasma insulin concentrations and metabolic and inflammatory variables. All blood samples were stored at –80 °C until final analyses were performed.

### Adipose tissue harvest

From the operating room: Fresh tissue samples (5–10 g) were processed within one hour upon procurement. After isolation of adipocytes and SVF^47^, ATMs and ARTs were isolated from SVF with biotinylated CD14 and CD3 antibodies, respectively (eBioscience and streptavidin-Dynabeads [Thermo Fisher Scientific]) or SVF was subjected to flow cytometry, as described in Supplementary Information. This methodology separates adipocytes, ATMs and ARTS (12), and the adequacy of separating the fractions is checked on every 10–15th sample obtained.

Liposuction: Abdominal SAT was obtained during the clamp procedure at baseline and 2 weeks after initiation of weight gain protocol. To obtain SAT, a skin incision (3–4 cm in length and 1 cm in depth) was made, and a small liposuction cannula was used to aspirate SAT under sterile conditions with local anesthesia (lidocaine 1% with 1:100,000 epinephrine). SAT was rapidly taken to the laboratory on ice to isolate adipocytes and prepare SVF for T cell flow analyses.

### Flow cytometry analyses

SVF cells were stained for CD4^+^CD25^+^Foxp3^+^ regulatory T cells (eBioscience Human Regulatory T Cell Staining Kit). Th1 (Tbet^+^ IFNG^+^) and Th2 (GATA3^+^) cells were stained with antibodies against CD4 (Clone RPA-T4, BD Pharmingen), CCR4 (Clone 1G1, BD Pharmingen), and CXCR3 (Clone REA232, Miltenyi Biotec)^48–50^. Dead cells were excluded using Fixable Viability Dye eFluor450 (eBioscience). Flow was performed on BD LSRII (BD Biosciences) or an Aurora (Cytek) spectral flow cytometer and analyzed with FlowJo (Tree Star) software. Whole adipose tissue was also analyzed by RT-PCR for CD3 and CD68 to assess T cell and macrophage infiltration, respectively. See Supplementary Information for flow cytometric standardization and gating strategy.

### Co-culture experiments

CD4 + T cells were isolated from PBMCs of obese patients with Miltenyi CD4 + T cell isolation kit. CD4 + T cells and corresponding VAT adipocyte cells were co-cultured in direct contact for four days before flow analyses for Th1 cells. Copaxone (glatiramer acetate, TEVA Pharmaceutical Industries) at 50 μg/ml was used as an antigen in the cultures.

### Adipocyte culture

After differentiation of human preadipocytes (Zen-Bio) into adipocytes according to the supplier’s protocol, cells were treated with recombinant IFNγ (2 ng/ml). RNA was collected after 24 hr. for expression of MHCII-related genes, adipokines/cytokines, and markers of mitochondrial function and fatty acid oxidation/synthesis (*n* = 3).

### Treg culture

1.5 × 10^6^ human Treg cells were isolated from 140cc peripheral blood, using the CD4 + CD25 + CD127^low^ kit (StemCell). Close to 50,000 cells/well in a 96-well plate were seeded and treated with human recombinant IFNγ (20 ng/ml). After 24 hr and 72 hr culture RNA samples were harvested. cDNA was amplified using the QuantiTech Whole Transcriptome kit (Qiagen) for gene expression analysis.

### Gene expression analyses

RNA was isolated from whole fat, primary adipocytes, ATMs and ARTs, human differentiated adipocytes (Zen-Bio), and cultured human blood Tregs using Direct-zol RNA Isolation kit (Zymo Research), reverse-transcribed with High-Capacity cDNA Reverse Transcription Kits (Thermo Fisher Scientific) and analyzed by quantitative real-time PCR (qPCR) using KiCqStart SYBR Green premade primers (Sigma) with expression values normalized to sample *PPIA* expression.

### Western blotting

Protein was isolated from human subcutaneous differentiated adipocytes (Zen-Bio) using RIPA lysis buffer, treated with recombinant IFNγ (R & D), IFNγ + JAK inhibitor (Sigma), or IFNγ + STAT1 siRNA (Dharmacon) and IFNγ + scrambled RNA and subjected to Western blotting performed as previously described^51^, using primary antibodies from Cell Signaling Technology: anti-phospho-STAT1 (Y701) (1:1000, 9171), anti-STAT1 (1:1000, 9172), and anti- MHCII (1:3000, 68258), from Abcam: anti-HLA-DPB1 (1:5000, ab157210), and from Santa Cruz: anti-β-actin (1:5000, sc-47778).

### STAT1 gene silencing

Gene silencing and IFNγ stimulation in differentiated subcutaneous adipocytes (Zen-Bio) was carried out between days 10–14 of differentiation by transfection with human STAT1 siRNA (5 µM, Dharmacon SMARTpool) using DharmaFECT transfection reagent. After a two-day transfection, recombinant human IFNγ (20 ng/ml) was added to the culture medium and continued for another two days before the samples were collected for western analysis.

### Seahorse bioanalyzer

Analyses was performed on primary adipocytes from lean and obese human SAT and VAT or on differentiated adipocytes with or without IFNγ (20 ng/mL). The oxygen-consumption rates (OCR; indicating mitochondrial respiration) and extracellular acidification rates (ECAR; indicating glycolysis) were monitored in a Seahorse XFe24 using the standard protocol of 3-min mix, 2-min wait and 3-min measure^52,53^. Oligomycin (a complex V inhibitor; 2 μM) was used to derive ATP-linked respiration and proton leak respiration. Carbonyl cyanide-p-trifluoromethoxy-phenyl-hydrazon (FCCP; 2 μM) was used to determine the cells maximal respiratory capacity by allowing the electron transport chain to function at its maximal rate. AntimycinA/Roteone (mitochondrial inhibitors; 0.5 μM) was used to determine nonmitochondrial respiration. Data from wells of the same treatment group were averaged together and analyzed directly using Waves software.

### Treg exhaustion analyses

Tregs were sorted from SVF and peripheral blood (PB) based on positive viability and the following markers: Lin-CD4 + CD25 + CD127dim using an Aria III (BD) FACS sorter. Cells were stained with antibodies against CD14 (HCD14; Catalog #325604), CD15 (W6D3; #323004), CD19 (HIB19; #302206), CD20 (2H7; #302304), CD56 (HCD56; #318304); TIGIT (A15153G; #372716); CD25 (M-A251; #356110); CD8 (SK1; #344724); CD127 (A019D5; #351332); CTLA-4 (BNI3; #369609); OX-40 (Ber-ACT35; #350012); PD-1 (EH12.2H7; #329949); CD4 (RPA-T4; #300530); CD3 (OKT3; #317330); Zombie Yellow Viability Stain all from BioLegend, with validation per the manufacturer’s website (https://www.biolegend.com/en-us/quality/quality-assurance-certificates). Additionally, cells were stained with Foxp3 (PCH101; #14-4776-82) from Invitrogen. Gating was determined based on staining antibody comparison to isotype control antibody.

To assess Treg function, previously separated PBMCs from the same patient were labeled with CellTrace CFSE (Invitrogen) and then cultured in the absence or presence of isolated blood or VAT Tregs (2 PBMC:1 Treg). Cells were cultured in a 96-well round bottom plate at 10,000 cells/well in the presence of one Treg Inspector Bead (Miltenyi) per 2 cells. After 4 days of culture, cells were stained for CD3 and viability and flow performed on an Aurora (Cytek).

### Quantification and statistical analyses

Statistical details of experiments can be found in the figure legends, including the statistical tests used, exact value of *n*, what *n* represents and dispersion and precision measures (e.g., mean, median, SD, SEM, confidence intervals). Data were examined for normality according to the Shapiro-Wilks criteria. Lean and obese groups were compared by student *T*-test for normally distributed variables and Mann-Whitney *U* test for non-normally distributed data. Pre- and post-surgery values were compared with paired *t*-tests. Pearson’s correlation for normally distributed and Spearman’s correlation for non-normally distributed variables were calculated to assess association. Multivariate linear regression analysis with independent variables of age, gender and BMI were calculated in stepwise fashion. Data were analyzed with SPSS Statistics for Windows, Version 24.0 (Armonk, NY). All samples reported were distinct samples and not repeated samples unless otherwise noted.

### Reporting summary

Further information on research design is available in the Nature Research Reporting Summary linked to this article.

## Supplementary information

Supplementary Information

Reporting Summary

## Data Availability

Source data are provided with this paper.

## Source data

Source Data
