# Supplementary material for: Interferon gamma mediates the reduction of adipose tissue regulatory T cells in human obesity
Source: Nat Commun. 2022 Sep 24;13:5606. doi: 10.1038/s41467-022-33067-5 (PMC9509397; doi:10.1038/s41467-022-33067-5)
Supplement: Supplementary file 1 — Supplementary Information [file 41467_2022_33067_MOESM1_ESM.pdf]

SUPPLEMENTARY INFORMATION

Methods

Flow cytometric standardization and gating strategy: Multiparameter flow cytometry was performed on SVF cells from adipose tissue using an LSRII (BD). We used BD Cytometer Setup and Tracking Beads (CST) to standardize voltages over time, and single-colored antibody-stained controls and an internal negative control population to establish gating. An isotype control antibody stained sample was used to determine negatives. SVF cells were gated on viability-dye negative cells to exclude dead cells, lymphogated on size to include lymphocytes, and doublets were excluded based on size (FSC) and granularity (SSC). Expression of CD4 was used to define T helper cells. Expression of CD25 and Foxp3 on T helpers defined regulatory T cells. Expression of $CXCR3^{+}CCR4^{-}$ defined Th1, and $CXCR3^{-}CCR4^{+}$ expression defined Th2 cells.

Analyses of Blood Samples: Plasma glucose, insulin, adiponectin and leptin concentrations were measured by enzyme-linked immunosorbent assays (Millipore). Insulin resistance was determined by the homeostasis model assessment of insulin resistance (HOMA-IR) and β-cell function was determined by HOMA-β [1].

Media and Buffers:

(1) **Collagenase Buffer:** HEPES (10mM), BSA (1%), and adenosine (50µM) in Gey's Balanced Salt Solution. Dissolve 50 mg collagenase II in 50 mL Collagenase Buffer for use.

(2) **Isolation Buffer:** DPBS w/o Ca^{2+} and Mg^{2+} containing 0.1% BSA and 5 mM EDTA.

(3) **Antibody Buffer**: DPBS w/o Ca^{2+} and Mg^{2+} containing 0.5% BSA and 5 mM EDTA

(4) **DMEM medium + FBS** (3%)

Isolation of adipose tissue fractions:

The fat tissue is dried, weighed, cleaned with DPBS without Ca^{2+} and Mg^{2+}, and minced and transferred to a 50-ml tube with Collagenase Buffer (1:1 in volume), then placed on Barnstead Max 4000 incubater at 180 rpm, 37°C for approximately 60 min. EDTA (final 10 mM) is then added with continued incubation for 5-10 min followed by addition of 20-30 ml of warm DMEM medium/3% FBS (20-30 ml) with gentle mixing and straining of the digested tissue on 300 and 500 µm cell strainers into a 50-ml tube, which is then in a Sorvall countertop Centrifuge (TX-1000 rotor) at 1100 rpm, 4°C for 8 min. The adipocyte layer is removed and washed and the SVF is isolated and prepared for flow analyses or Dynabead collection of the ATMs and ARTs.

10. SVF (from Step 6B): Add 30 ml Isolation Buffer to the 50-ml tube with SVF in the LCK lysing buffer, filter the cells with a pre-wet 70 µm strainer laying on top of a 50 ml tube. Spin down the tube with cells at 1,300 rpm (400 x g), 4°C for 6 min. (SVF cells may be stored at 4°C at this point until next day by adding FACS Buffer)

11. SVF (for macrophage and then T cells): Discard supernatant,

- 11a): If cells will be used for flow cytometry, add 2 ml of FACS Buffer, mix and transfer to a 2-ml tube and follow the “T Cell Flow Protocol”, or
- 11b): If no flow, just add 500 µl of cold Antibody Buffer, and pipet and gently vortex to resuspend cells well without cell clusters. Transfer to a fresh 2-ml tube. Add 10 µl of mouse F4/80 biotin antibody (use CD14 biotin antibody for human samples), and incubate at 4°C for 30 min on a rocking rack.

12. SVF (for macrophage): Add 1.5 ml cold Isolation Buffer to wash cells and centrifuge at 400 x g for 6 min at 4°C. Remove and discard the supernatant. Add the 1 ml Isolation Buffer containing 40 µl pre-washed beads to the pellet and mix well. Transfer all content to a flow tube. Incubate for 20 min at 4°C on a slow rotating rack.

13. Place the flow tube containing SVF on magnet rack for 2 min. While the flow tube is still in the magnet, carefully transfer all bead-free supernatant containing T cells into a 15 ml tube.

14. Remove the flow tube from the magnet and add 1 ml of cold Isolation Buffer, pipet several times and place the flow tube on magnet rack for 2 min. Repeat Step 13-14 twice to wash off non-specific binding including T cells. Collect all washed buffer into the 15 ml tube for Step 16.

15. SVF (for macrophage): Remove the flow tube from the magnet. Add 300 µl of Zymo TRI Reagent (or 500 µl of Trizol) to the beads linked to ATM and transfer all together into a 1.5 ml tube, vortex well, and store it in -80°C freezer for RNA extraction.

16. SVF (for T-cell): Spin the 15 ml tube from Step 15, at 1,300 rpm, 4°C for 7 min. Discard supernatant (*The pellet should be evident*). Add 500 µl of Antibody Buffer to the pellet, and pipet and gently vortex to resuspend cells well without cell clusters. Transfer to a fresh 2-ml tube. Add 10 µl of CD3e biotin antibody, mix well and incubate at 4°C for 30 min on a gentle rocking rack.

17. ART: Add 1.5 ml cold Isolation Buffer to wash cells and centrifuge at 500g for 5 min at 4°C. Remove and discard the supernatant. Add the 1 ml Isolation Buffer containing 40µl pre-washed beads to the anti-CD3e-coated cell pellet and mix well. Transfer all content to a flow tube. Incubate for 20 min at 4°C on a slow rotating rack.

18. ART: Set the flow tube on magnet rack for 2 min. While the flow tube is still in the magnet, carefully discard the liquid.

19. Remove the flow tube from the magnet and add 1 ml of cold Isolation Buffer, pipet several times and place the flow tube on magnet rack for 2 min. Repeat Step 18-19 twice to wash off and discard non-specific binding cells.

20. Finally, Add 300 µl of Zymo TRI Reagent (or 500 µl of Trizol) to the beads linked to ART and transfer all together into a 1.5 ml tube, vortex well, and store it in -80°C freezer for RNA extraction.

Supplementary Table 1. SVF cells yield for flow cytometry and RN.

Mean				
VAT weight (g)	Total VSVF cell #		SAT weight (g)	Total SSVF cell #
4.55	2,091,961		2.02	473,542
cells/g tissue=	459,534		cells/g tissue=	234,523

General Laboratory Operation

a) Laboratory Procedure Standardization: These studies (or specific parts thereof) were performed using established laboratory protocols.

b) Status of Assay Qualification and Validation: These studies were performed using validated assays.

Supplementary Results

Raw data can be provided per request. Median and ranges of the event counts for the most relevant cell populations acquired:

Lean Subcutaneous Adipose Tissue (SAT):

Regulatory T Cells (Tregs): Median 21.7%; Minimum 4.5%; Maximum 41.7%

T helper (Th) Type 1 Cells: Median 36.2%; Minimum 0.0%; Maximum 63.9%

T helper (Th) type 2 Cells: Median: 18.7%; Minimum: 3.6%; Maximum: 56.1%

Obese Subcutaneous Adipose Tissue (SAT):

Regulatory T Cells (Tregs): Median 5.8%; Minimum 0.8%; Maximum 16.1%

T helper (Th) Type 1 Cells: Median 29.0%; Minimum 4.8%; Maximum 80.8%

T helper (Th) type 2 Cells: Median 17.0%; Minimum 3.9%; Maximum 54.1%

Lean Visceral Adipose Tissue (VAT):

Regulatory T Cells (Tregs): Median 12.6%; Minimum 3.3%; Maximum 51.6%

T helper (Th) Type 1 Cells: Median 57.8%; Minimum 4.7%; Maximum 63.5%

T helper (Th) type 2 Cells: Median 10.2%; Minimum 0.0%; Maximum 41.3%

Obese Visceral Adipose Tissue (VAT):

Regulatory T Cells (Tregs): Median 3.0%; Minimum 0.2%; Maximum 13.0%

T helper (Th) Type 1 Cells: Median 46.4%; Minimum 1.0%; Maximum 90.3%

T helper (Th) type 2 Cells: Median 9.3%; Minimum 0.5%; Maximum 40.3%.

Supplementary Table 2. Correlation Coefficients (r) Between Adipocyte Gene Expression (Fold Change) of MHCII genes in Visceral (VAT) and Subcutaneous (SAT) Adipose Tissue and Markers of Mitochondrial Function, Fatty Acid β-oxidation/synthesis, and Inflammation. Correlations were two-sided, without correction for multiple comparisons.

	**VAT Adipocyte MHCII Gene Expression**				**SAT Adipocyte MHCII Gene Expression**			
	**CIITA**	**HLA-DPA1**	**CD74**	**CD80**	**CIITA**	**HLA-DPA1**	**CD74**	**CD80**
Mitochondrial Function Genes								
**CIDEA**	**r= -0.568***	r= -0.110	**r= -0.375***	**r= -0.493***	**r= -0.648***	r= -0.073	r= -0.133	**r= -0.571***
**ATP5A**	**r= -0.784***	r= +0.009	**r= -0.512***	**r= -0.794***	**r= -0.597***	r= -0.063	**r= -0.566***	**r= -0.719***
**CPT1B**	**r= -0.585***	r= +0.063	**r= -0.413***	**r= -0.533***	**r= -0.709***	r= +0.061	**r= -0.818***	**r= -0.700***
**COX5A**	r= -0.257	r= +0.200	**r= -0.600***	r= +0.000	**r= -0.320***	r= -0.037	r= -0.198	r= -0.022
Fatty Acid β-Oxidation Gene								
**ACADM**	**r= -0.712***	r= -0.065	**r= -0.481***	**r= -0.686***	**r= -0.612***	**r= -0.340***	**r= -0.553***	**r= -0.687***
Fatty Acid Synthesis Genes								
**ACC2**	**r= -0.625***	r= -0.023	**r= -0.325***	**r= -0.648***	**r= -0.722***	**r= -0.091**	**r= -0.396***	**r= -0.761***
**FASN**	**r= -0.534***	r= -0.124	**r= -0.334***	**r= -0.532***	**r= -0.291**	**r= -0.374***	**r= -0.402***	**r= -0.274**
**DGAT**	**r= -0.728***	r= +0.012	**r= -0.429***	**r= -0.658***	**r= -0.378***	r= -0.025	**r= -0.467***	**r= -0.364***
Pro-Inflammatory Cytokine Genes								
**LEPTIN**	**r= -0.497***	r= +0.114	r= -0.163	**r= -0.274***	**r= -0.522***	r= +0.015	r= -0.169	**r= -0.514***
**IL-1β**	**r= +0.379***	**r= +0.456***	r= -0.124	r= -0.122	r= +0.210	**r= +0.620***	r= +0.213	r= -0.014
**TNFα**	r= -0.112	**r= +0.503***	r=+0.059	r=-0.095	**r = +0.318***	**r= +0.686***	**r= +0.425***	r= +0.191

**NLRP3**	r= -0.157	**r= +0.470***	r= -0.032	r= -0.012	**r = +0.391***	**r= +0.743***	**r= +0.287***	r= -0.078
**PAI-1**	**r- +0.620**	r= +0.001	**r= +0.469***	**r= +0.342***	**r= +0.432***	**r= +0.589***	**r= +0.526***	**r= +0.361***
Anti-Inflammatory Cytokine Genes								
**ADIPOQ**	**r= -0.750***	r= +0.037	**r= -0.287***	**r= -0.622***	**r= -0.658***	r= -0.005	**r= +0.296***	**r= -0.724***
**PPARG**	**r= -0.424***	r= +0.005	**r= -0.307***	**r= -0.585***	**r= -0.623***	r= -0.089	**r= -0.330***	**r= -0.802***

Abbreviations: CD: Cluster of differentiation; HLA: Human leukocyte antigen complex; CIITA: class II major histocompatibility complex transactivator; ADIPOQ: adiponectin; CIDEA: cell death activator; ATP5A: ATP synthase 5A; CPT1B: carnitine palmitoyltransferase 1B; ACADM: Acetyl CoA dehydrogenase; ACC2: acetyl-CoA carboxylase; FASN: fatty acid synthase; DGAT: diglyceride acyltransferase; Significant correlations are bolded; *, $p < 0.05$.

Supplementary Table 3. Correlation Coefficients (r) Between Adipocyte Gene Expression (Fold Change) in Visceral (VAT) and Subcutaneous (SAT) Adipose Tissue and Body Mass Index (BMI). Correlations were two-sided, without correction for multiple comparisons.

	**VAT Gene Expression**	**SAT Gene Expression**
Major Histocompatibility 2 (MHCII) genes		
CIITA	r=+0.116	r=+0.495*
HLA-DPA1	r=+0.084	r=+0.326*
CD74	r=-0.012	r=+0.349*
CD80	---	r=+0.348*
Mitochondrial Function Genes		
CIDEA	r=-0.182	r=-0.517*
ATP5A	r=-0.119	r=-0.342*
CPT1B	r=-0.134	r=-0.548*
Fatty Acid Beta Oxidation Genes		
ACADM	r=-0.317*	r=-0.527*
Fatty Acid Synthesis Genes		
ACC2	r=-0.191	r=-0.467*
FASN	r=-0.217*	r=-0.481*
DGAT	r=-0.087	r=- 0.388*
Pro-inflammatory Mediator Gene Expression		
IL-1B	r=-0.028	r=+0.465*
Leptin	r=+0.175	r=+0.259*
NLRP3	r=+0.073	r=+0.426*
TNFA	r=-0.138	r=+0.523*
Anti-inflammatory Mediator Gene Expression		
PPARG	r=-0.323*	r=-0.350*

ADIPOQ	r=-0.241*	r =-0.368*

Abbreviations: CD: Cluster of differentiation; HLA: Human leukocyte antigen complex; CIITA: class II major histocompatibility complex transactivator; ADIPOQ: adiponectin; CIDEA: cell death activator; ATP5A: ATP synthase 5A; CPT1B: carnitine palmitoyltransferase 1B; ACADM: Acetyl CoA dehydrogenase; ACC2: acetyl-CoA carboxylase; FASN: fatty acid synthase; DGAT: diglyceride acyltransferase; PPARG: PPARgamma; Nucleotide-Binding Oligomerization Domain, Leucine Rich Repeat And Pyrin Domain Containing 3 (NLRP3); TNFA: tumor necrosis factor alpha; IL: Interleukin; PAI-1:plasminogen activator inhibitor-1. *, $p < 0.05$.

Supplementary Table 4. Significant Correlation Coefficients (r) Between Obese Adipocyte Gene Expression (Fold Change) of MHCII genes in Visceral (VAT) and Subcutaneous (SAT) Adipose Tissue and Markers of Mitochondrial Function, Fatty Acid β-oxidation/synthesis after exclusion of lean subjects. Correlations were two-sided, without correction for multiple comparisons.

	CIITA	CD74	CD80	CIITA	CD74	CD80
Mitochondrial Function Genes						
**CIDEA**	r= -0.568*			r= -0.515*		r= -0.441*
**ATP5A**	r= -0.656*	r= -0.396*	r= -0.656*	r= -0.576*	r= -0.597*	r= -0.752*
**CPT1B**	r= -0.416*	r= -0.305*	r= -0.512*	r= -0.731*	r= -0.317[ǂ]	r= -0.645*
**COX5A**		r= -0.600*		r= -0.694*	r= -0.694*	
Fatty Acid β-Oxidation Gene						
**ACADM**	r= -0.577*	r= -0.481*	r= -0.627*		r= -0.434*	r= -0.661*
Fatty Acid Synthesis Genes						
**ACC2**	r= -0.453*	r= -0.352*	r= -0.580*	r= -0.499*		r= -0.682*
**FASN**	r= -0.501*	r= -0.284[ǂ]	r= -0.628*	r= -0.291		
**DGAT**	r= -0.571*	r= -0.333*	r= -0.481*	r= -0.571[ǂ]	r= -0.441[ǂ]	r= -0.485*

Abbreviations: CD: Cluster of differentiation; CIITA: class II major histocompatibility complex transactivator; CIDEA: cell death activator; ATP5A: ATP synthase 5A; CPT1B: carnitine palmitoyltransferase 1B; ACADM: Acetyl CoA dehydrogenase; ACC2: acetyl-CoA carboxylase; FASN: fatty acid synthase; DGAT: diglyceride acyltransferase. *, $p < 0.05$, [ǂ] $p < 0.10$.

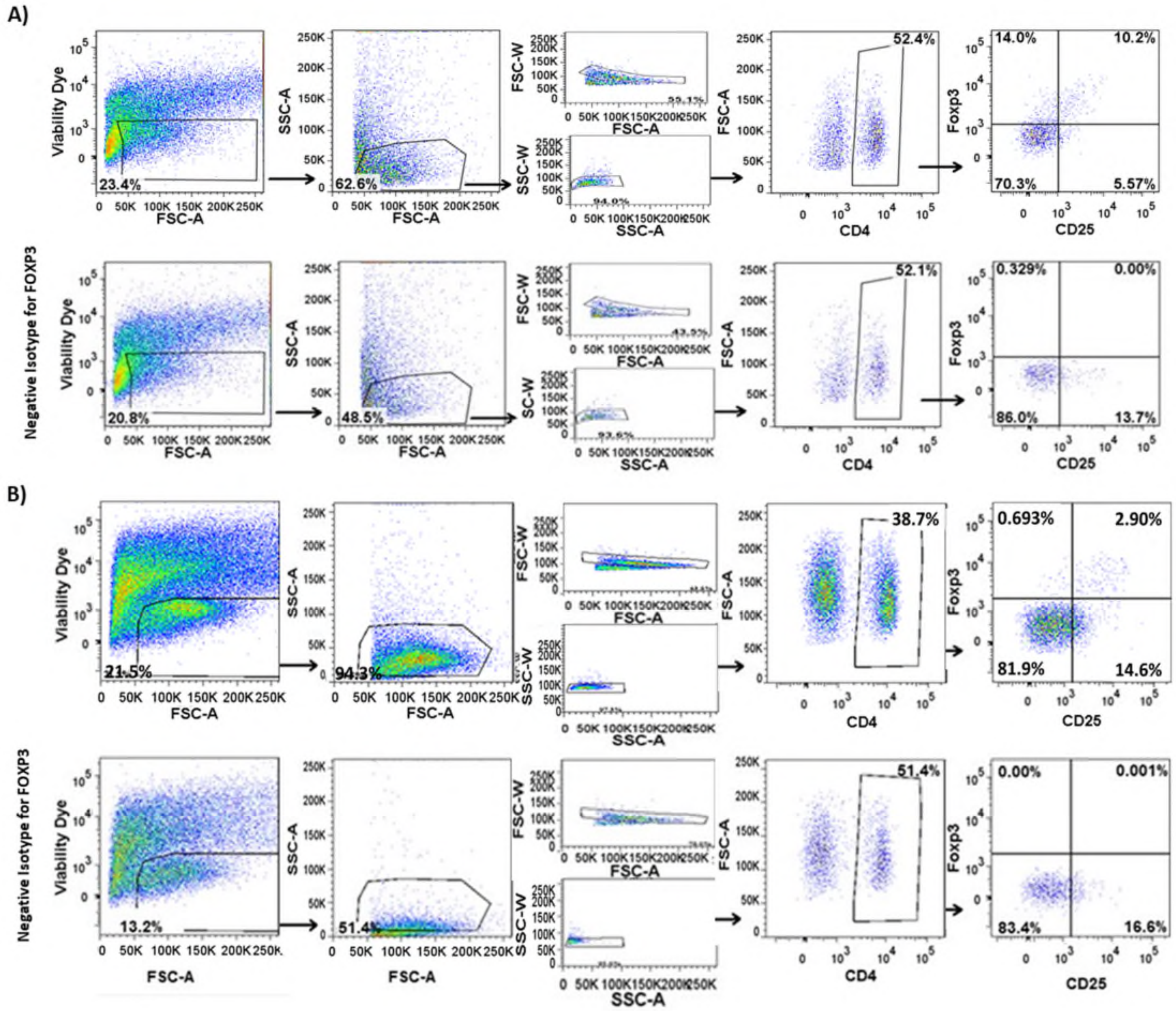

Supplementary Figure 1. Representative plots for flow cytometry and FOXP3 isotype controls from a lean (**A**) and an obese (**B**) human subject.

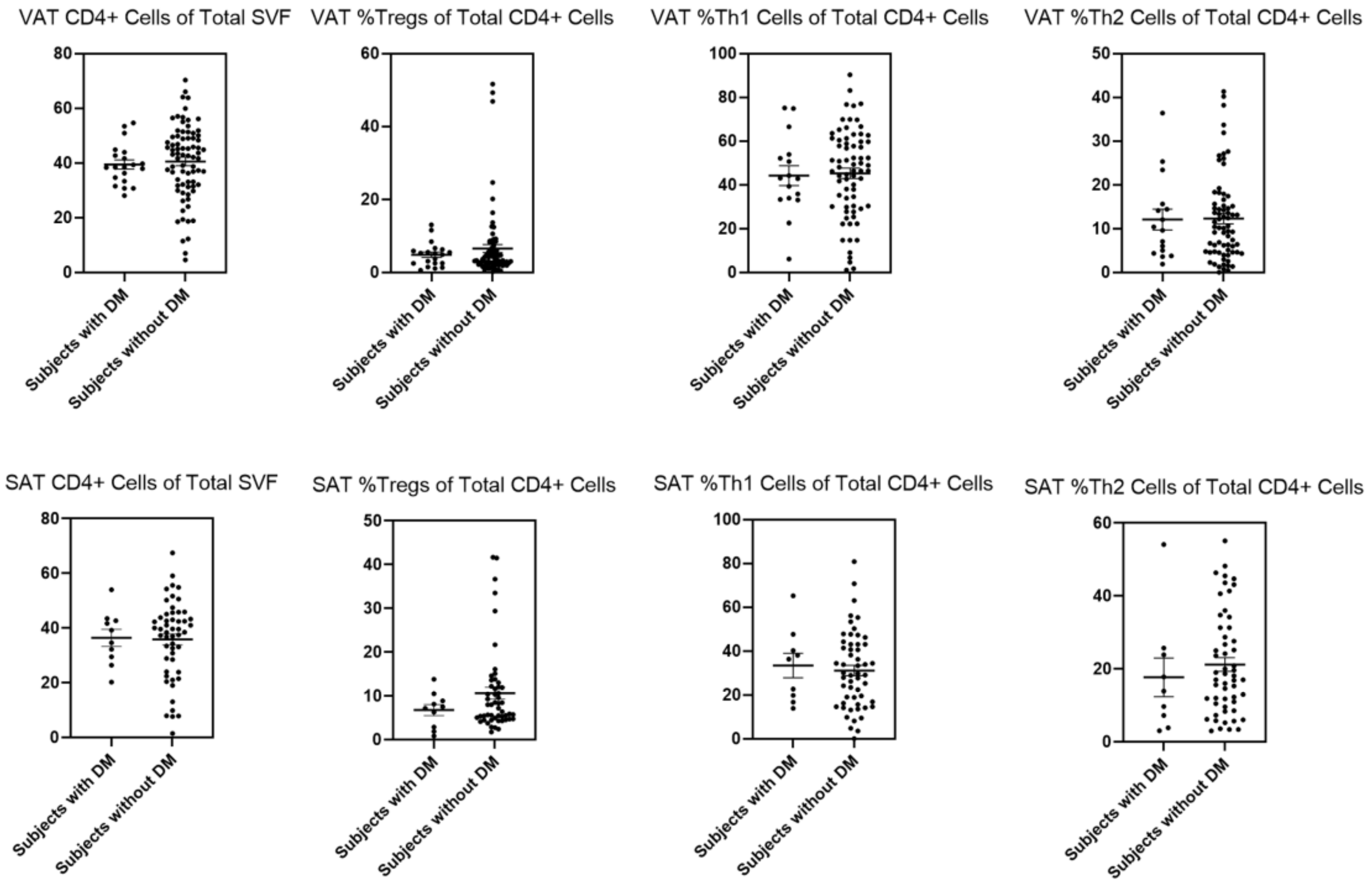

Supplementary Figure 2. T lymphocytes in subcutaneous adipose tissue (SAT) and visceral adipose tissue (VAT) in patients with (n=20 biologically independent subjects) and without diabetes (n=98 biologically independent subjects) analyzed by two-sided, independent t-test. No replicates were included in data analyses. Data presented as mean ± SEM.

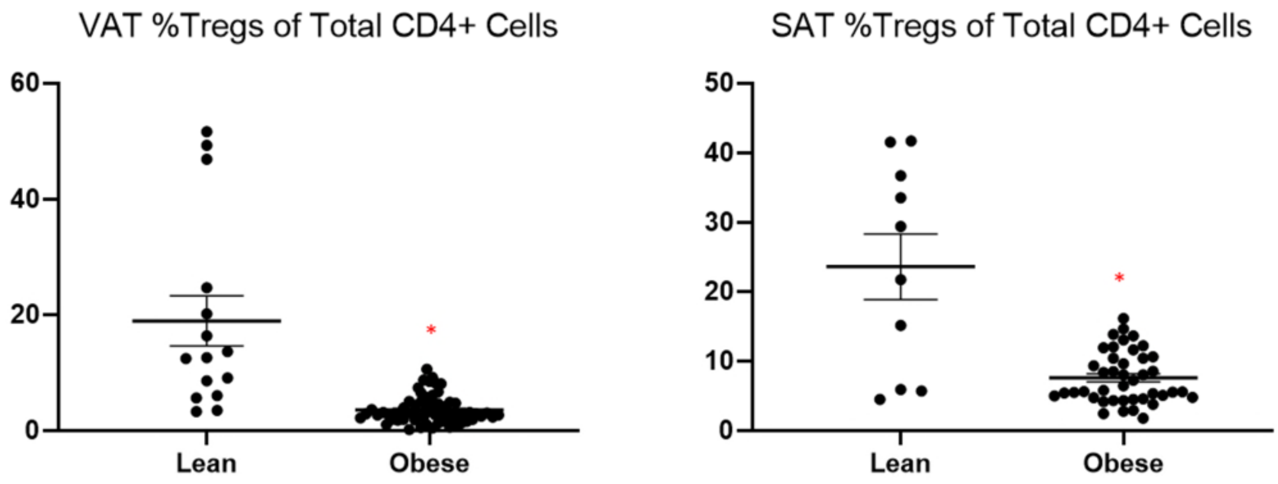

Supplementary Figure 3. Adipose regulatory T (Treg) lymphocytes as percent (%) of total CD4+ cells in A) visceral adipose tissue (VAT) and B) subcutaneous adipose tissue (SAT) in a subset of patients without type 2 diabetes (n= 78 biologically independent subjects) analyzed by two-sided independent sample T-test, $P<0.05$. Data presented as mean ± SEM.

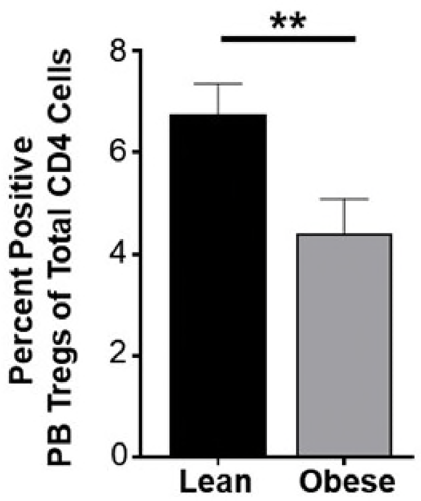

Supplementary Figure 4. Regulatory T (Treg) lymphocytes as percent (%) of total CD4+ cells in peripheral blood (PB) by flow cytometry of lean (n=13) and obese (n=12) patients analyzed by two-sided independent sample T-test. Data presented as mean ± SEM.

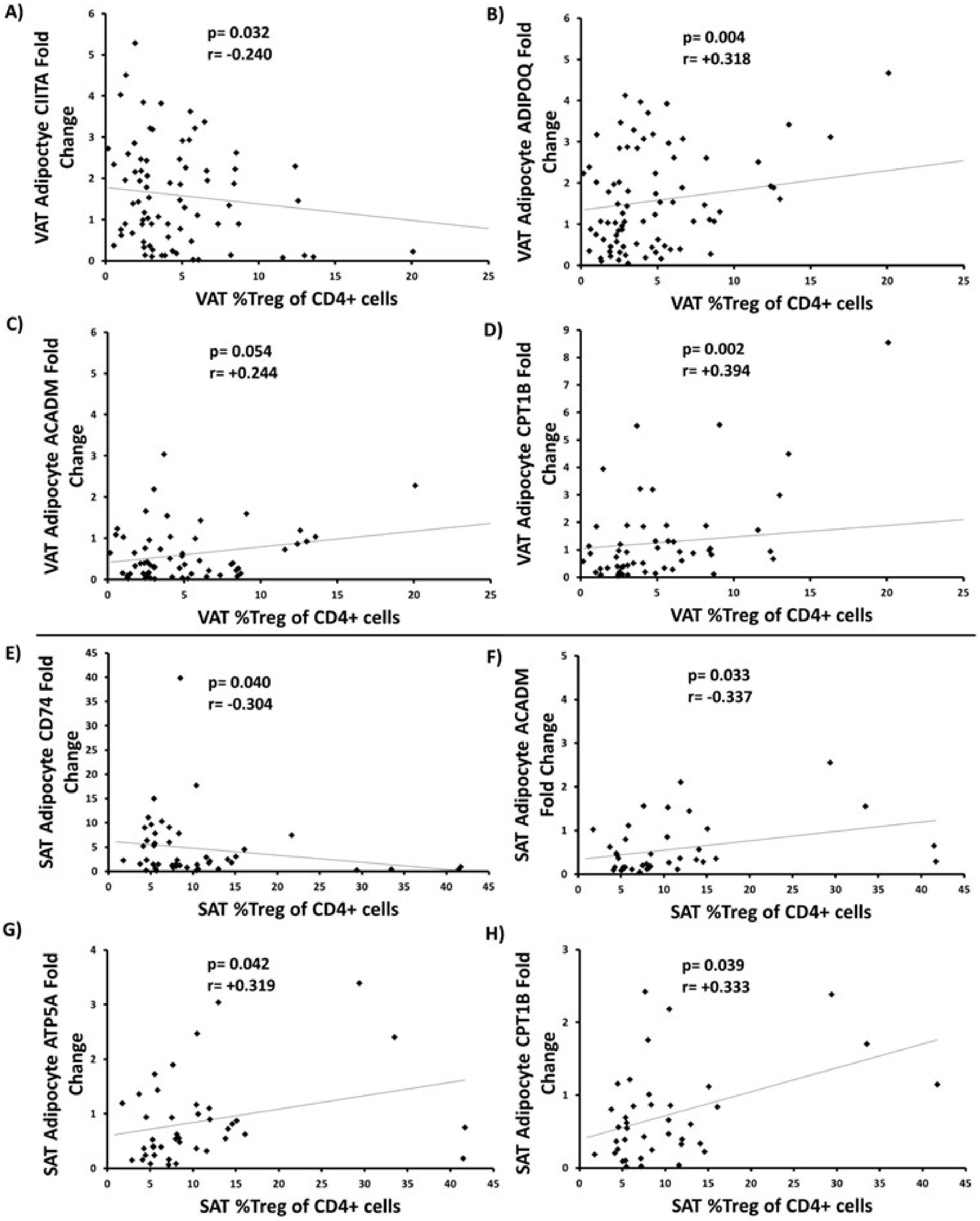

Supplementary Figure 5. Correlations (Spearman/Pearson) between (**A-D**) visceral (VAT) and (**E-H**) subcutaneous (SAT) adipocyte gene expression and adipose regulatory T (Treg) cells as percent (%) of total CD4+ cells. Correlations were two-sided, without correction for multiple comparisons.

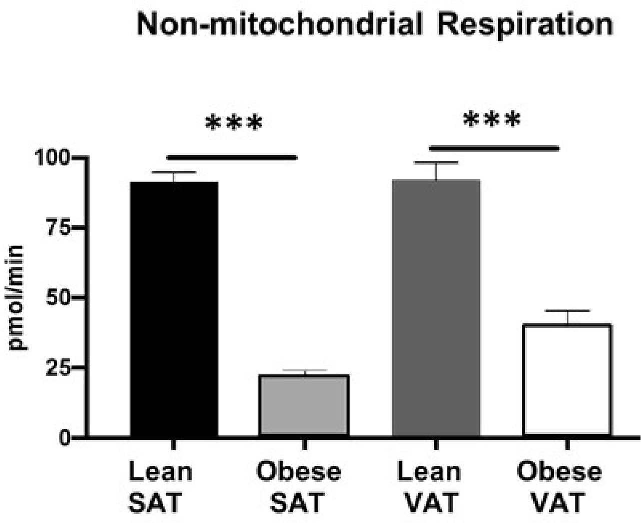

Supplementary Figure 6. Non-mitochondrial respiration in human adipocytes from lean and obese human subcutaneous (SAT) (n=2) and visceral (VAT) (n=3) white adipose tissue by Seahorse. Data presented as mean ± SEM. ***, $p<0.001$.

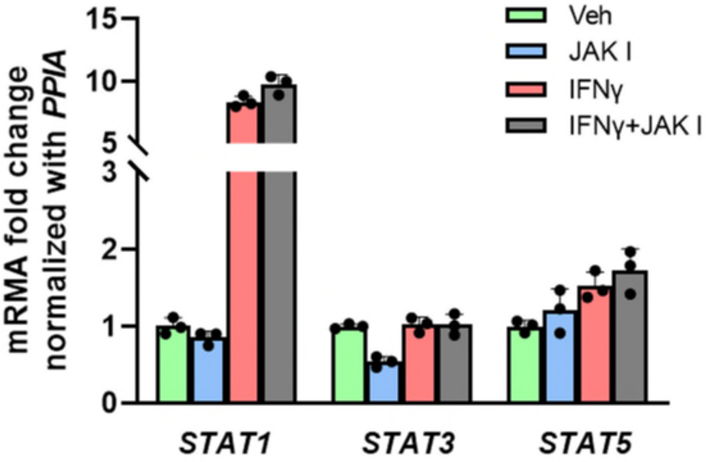

Supplementary Figure 7. Gene expression levels of *STAT1*, *STAT3,* and *STAT5* in mature subcutaneous adipocytes treated with JAK inhibitor (JAK I, 10 uM) with or without IFNγ (20ng/ml) for 48 hr. (N = 3). Data presented as mean ± SEM.

Supplementary References

1. Matthews, D.R., *et al.* Homeostasis model assessment: insulin resistance and beta-cell function from fasting plasma glucose and insulin concentrations in man. *Diabetologia* **28**, 412-419 (1985).
