# Supplementary material for: Interferon gamma mediates the reduction of adipose tissue regulatory T cells in human obesity
Source: Nat Commun. 2022 Sep 24;13:5606. doi: 10.1038/s41467-022-33067-5 (PMC9509397; doi:10.1038/s41467-022-33067-5)
Supplement: Supplementary file 2 — Reporting Summary [file 41467_2022_33067_MOESM2_ESM.pdf]

nature portfolio

Corresponding author(s): David Bradley, MD, MS and Willa A. Hsueh, MD

Last updated by author(s): 8/13/22

Reporting Summary

Nature Portfolio wishes to improve the reproducibility of the work that we publish. This form provides structure for consistency and transparency in reporting. For further information on Nature Portfolio policies, see our Editorial Policies and the Editorial Policy Checklist.

Statistics

For all statistical analyses, confirm that the following items are present in the figure legend, table legend, main text, or Methods section.

n/a	Confirmed	
☐	☒	The exact sample size (*n*) for each experimental group/condition, given as a discrete number and unit of measurement
☐	☒	A statement on whether measurements were taken from distinct samples or whether the same sample was measured repeatedly
☐	☒	The statistical test(s) used AND whether they are one- or two-sided<br>*Only common tests should be described solely by name; describe more complex techniques in the Methods section.*
☐	☒	A description of all covariates tested
☐	☒	A description of any assumptions or corrections, such as tests of normality and adjustment for multiple comparisons
☐	☒	A full description of the statistical parameters including central tendency (e.g. means) or other basic estimates (e.g. regression coefficient) AND variation (e.g. standard deviation) or associated estimates of uncertainty (e.g. confidence intervals)
☐	☒	For null hypothesis testing, the test statistic (e.g. *F*, *t*, *r*) with confidence intervals, effect sizes, degrees of freedom and *P* value noted<br>*Give P values as exact values whenever suitable.*
☒	☐	For Bayesian analysis, information on the choice of priors and Markov chain Monte Carlo settings
☒	☐	For hierarchical and complex designs, identification of the appropriate level for tests and full reporting of outcomes
☐	☒	Estimates of effect sizes (e.g. Cohen's *d*, Pearson's *r*), indicating how they were calculated

Our web collection on statistics for biologists contains articles on many of the points above.

Software and code

Policy information about availability of computer code

Data collection	No software was used
Data analysis	Data were analyzed with SPSS Statistics for Windows, Version 24.0 (Armonk, NY).

For manuscripts utilizing custom algorithms or software that are central to the research but not yet described in published literature, software must be made available to editors and reviewers. We strongly encourage code deposition in a community repository (e.g. GitHub). See the Nature Portfolio guidelines for submitting code & software for further information.

Data

Policy information about availability of data

All manuscripts must include a data availability statement. This statement should provide the following information, where applicable:

- Accession codes, unique identifiers, or web links for publicly available datasets
- A description of any restrictions on data availability
- For clinical datasets or third party data, please ensure that the statement adheres to our policy

Access to the minimum dataset that are necessary to interpret, verify and extend the research in the article, transparent to readers will be granted upon request.

Field-specific reporting

Please select the one below that is the best fit for your research. If you are not sure, read the appropriate sections before making your selection.

☒ Life sciences ☐ Behavioural & social sciences ☐ Ecological, evolutionary & environmental sciences

For a reference copy of the document with all sections, see nature.com/documents/nr-reporting-summary-flat.pdf

Life sciences study design

All studies must disclose on these points even when the disclosure is negative.

Sample size	Sample size was determined based on our preliminary data (n=5) which showed a decrese in adipose tissue regulatory T cells (Tregs) (4.6 +/- 2.4% vs. 2.3 +/-1.1%) from before to after 2 weeks of hypercaloric, high-fat diet. With n=10 subjects, we estimated >85% power to detect a difference inTregs with α=0.05.
Data exclusions	No data were excluded in the analyses.
Replication	When possible, all attempts at replication were successful.
Randomization	Subjects were not randomized in the included studies.
Blinding	Blinding was not performed in the included studies.

Reporting for specific materials, systems and methods

We require information from authors about some types of materials, experimental systems and methods used in many studies. Here, indicate whether each material, system or method listed is relevant to your study. If you are not sure if a list item applies to your research, read the appropriate section before selecting a response.

Materials & experimental systems

n/a	Involved in the study	
☐	☒	Antibodies
☒	☐	Eukaryotic cell lines
☒	☐	Palaeontology and archaeology
☒	☐	Animals and other organisms
☐	☒	Human research participants
☒	☐	Clinical data
☒	☐	Dual use research of concern

Methods

n/a	Involved in the study	
☒	☐	ChIP-seq
☐	☒	Flow cytometry
☒	☐	MRI-based neuroimaging

Antibodies

Antibodies used	For flow analysis of T cell exhaustion markers, SVF and peripheral blood cells were stained as described above. Cells were stained with antibodies against the following CD14 (HCD14), CD15 (W6D3), CD19 (HIB19), CD20 (2H7), CD56 (HCD56); TIGIT (A15153G); CD25 (M-A251); CD8 (SK1); CD127 (A019D5); CTLA-4 (BNI3); OX-40 (Ber-ACT35); PD-1 (EH12.2H7); CD4 (SK3); CD3 (OKT3); Zombie Yellow Viability Stain all from BioLegend. Additionally, cells were stained with Foxp3 (PCH101) from Invitrogen.
Validation	Validation was performed by the manufacturer's which can be found readily on the manufacturer's websites.

Human research participants

Policy information about studies involving human research participants

Population characteristics	Full Details of population characteristics are listed in Table 1.
Recruitment	Paitents were recrutied directly from the Center for MInimally Invasive Surgery (main study cohort) or through the use of approved flyers (weight gain cohort).
Ethics oversight	The Ohio State University Institutional Review Board

Note that full information on the approval of the study protocol must also be provided in the manuscript.

Clinical data

Policy information about clinical studies

All manuscripts should comply with the ICMJE guidelines for publication of clinical research and a completed CONSORT checklist must be included with all submissions.

Clinical trial registration	N/A The collection of tissues and blood and the weight gain study, none of which are clinical trials, are not listed with Clinicaltrials.gov. The NIH defines a clinical trial as a research study in which one or more human subjects are prospectively assigned to one or more interventions (which may include placebo or other control) to evaluate the effects of those interventions on health-related biomedical or behavioral outcomes. We were not testing a dietary intervention comparing diets or treatment.
Study protocol	Surgical Study Population. VAT and SAT biopsies were obtained from obese (n=83, age 45.0 ± 11.3 yo, BMI 48.8 ± 8.5 kg/m2) and lean (n=15, age 42.9 ± 12.3 yo, BMI 23.6 ± 1.8 kg/m2) patients who underwent elective surgery at The Ohio State University (OSU) Center for Minimally Invasive Surgery (Table 1). Elective surgery in the lean subjects consisted of 6 cholecystectomies, 6 hernia repairs, 2 Nissen fundoplications, and 1 myotomy. Elective bariatric surgery in the obese group consisted of 47 subjects undergoing sleeve gastrectomy and 36 undergoing Roux-en-Y gastric bypass. The study was approved by The OSU Institutional Review Board (IRB), and all participants provided written informed consent.<br><br>Weight gain protocol. A separate group of lean metabolically healthy patients (n=11, age 27.0 ± 7.0 yo, baseline BMI 22.4 ± 1.8 kg/m2) were instructed by a registered dietician (RD) to consume at minimum an additional 1,320 kcal/day with >50% of total caloric intake in total fat and >10% in saturated fats by dining at fast-food restaurants (using meal vouchers) combined with a high calorie liquid formula, TwoCal® HN. TwoCal® HN is a nutritionally complete formula available in two flavors and provides 475 calories per 8 fluid ounces. Subjects were instructed not to change their level of physical activity or start any new medications during the study. The study was approved by The OSU Institutional Review Board (IRB), and all participants provided written informed consent. All study team members were certified by the Collaborative Institutional Training Initiative (CITI Program) throughout the study period, including training in Human Subjects Research and Good Clinical Practice.<br>The subjects were followed closely by the RD, who advised subjects on what to eat when dining and also incorporate TwoCal® HN supplements into their individualized meal plans, to assist in matching the rate of weight gain. After completion of the weight gain protocol, each participant met individually with the RD to plan a hypocaloric diet, based on their food records, to facilitate losing the weight gained, if desired.<br><br>Full study protocols are available in the submitted manuscript.
Data collection	We recruited the obese study subjects through the Center for Minimally Invasive Surgery at The Ohio State University Wexner Medical Center and lean subjects using flyers and ads around campus. We asked potential participants if they might have an interest in participating and if so, we collected a phone number so that a co-PI or a research team member could follow-up with a telephone call to the potential participant approximately one week later. The research team also provided contact information to the participant so that the potential participant may call with any questions, concerns, etc. The PI or a research team member scheduled the screening study visit during the follow-up phone call if individuals or their designated representatives were interested in participating.<br>A trained member of the research team verbally reviewed the informed consent document in its entirety at the screening visit before any study procedures took place. This review was in a private setting at one of the Clinical Research Center (CRC) units at The Ohio State University Wexner Medical Center. We encouraged participants and/or a legal representative to ask questions throughout the review of the document as well as at the completion of reviewing the document. The research team used teach-back and paraphrasing techniques to ensure the participant understood their role and responsibility as part of this research study. After addressing all questions and concerns, we asked participants interested in moving forward with the study to sign the informed consent document. After the participant and a member of the research team signed the document, we made two copies of the document - one for the participant and one for the CRC unit.
Outcomes	The primary outcomes were the change in adipose tissue regulatory T cells (Tregs) from before to after 2 weeks of hypercaloric, high-fat diet and the difference between lean and obese human subjects in adipose tissue Treg abundance.

Flow Cytometry

Plots

Confirm that:

☒ The axis labels state the marker and fluorochrome used (e.g. CD4-FITC).

☒ The axis scales are clearly visible. Include numbers along axes only for bottom left plot of group (a 'group' is an analysis of identical markers).

☒ All plots are contour plots with outliers or pseudocolor plots.

☒ A numerical value for number of cells or percentage (with statistics) is provided.

Methodology

Sample preparation	Isolation of adipocyte, ATM and ARTs from human AT. Fresh tissue samples (10 g) were processed within one hour upon procurement. After isolation of adipocytes and SVF (7), ATMs and ARTs were isolated from SVF with biotinylated CD14 and CD3 antibodies, respectively (eBioscience and streptavidin-Dynabeads [Thermo Fisher Scientific]) or SVF was subjected to flow cytometry, as described in Supplementary Material.

	Complete isolation procedure is documented in Supplementary Material.
Instrument	Flow was performed on BD LSRII (BD Biosciences) or an Aurora (Cytek) spectral flow cytometer and analyzed with FlowJo (Tree Star) software.
Software	Flow was performed on BD LSRII (BD Biosciences) or an Aurora (Cytek) spectral flow cytometer and analyzed with FlowJo (Tree Star) software.
Cell population abundance	Cell abundances are detailed in Supplementary Material.
Gating strategy	Flow cytometric standardization and gating strategy: Multiparameter flow cytometry was performed on SVF cells from adipose tissue using an LSRII (BD). We used BD Cytometer Setup and Tracking Beads (CST) to standardize voltages over time, and single-colored antibody-stained controls and an internal negative control population to establish gating. An isotype control antibody stained sample was used to determine negatives. SVF cells were gated on viability-dye negative cells to exclude dead cells, lymphogated on size to include lymphocytes, and doublets were excluded based on size (FSC) and granularity (SSC). Expression of CD4 was used to define T helper cells. Expression of CD25 and Foxp3 on T helpers defined regulatory T cells. Expression of CXCR3+CCR4- defined Th1, and CXCR3-CCR4+ expression defined Th2 cells.

☒ Tick this box to confirm that a figure exemplifying the gating strategy is provided in the Supplementary Information.
